# A Keratinocyte‐Mast Cell NF‐κB2/CXCL2/IL‐6 Amplification Loop Enhances Cutaneous Antifungal Defense Against *C. albicans*

**DOI:** 10.1002/advs.202520409

**Published:** 2026-05-04

**Authors:** Yan Yuan, Manyun Mao, Jiaan Zhang, Zhimin Duan, JiaNing Wang, Yujie Chen, Sihan Chen, Chuting Liang, Xvyue Zhou, Songmei Geng, Min Li, Xu Chen, Ni Lian

**Affiliations:** ^1^ Jiangsu Provincial Key Laboratory of Dermatology Institute of Dermatology Hospital For Skin Diseases Chinese Academy of Medical Sciences & Peking Union Medical College Nanjing China; ^2^ Department of Dermatology The Second Affiliated Hospital of Xi'an Jiaotong University Xi'an China; ^3^ Center For Global Health School of Public Health Nanjing Medical University Nanjing China

**Keywords:** amplification loop, *C. albicans* infection, keratinocytes, mast cells

## Abstract

Mast cells (MCs), key innate immune sentinels at the host–environment interface, serve as primary responders to invading pathogens. However, their specific contribution to host defense against cutaneous *Candida albicans* (*C. albicans*) infection and their synergy with other immune and non‐immune cells remain poorly understood. Here, we show that MCs accumulate locally in skin lesions of patients with cutaneous candidiasis. In mouse models, MC‐deficient Kit^W‐sh/W‐sh^ mice developed more severe infection than wild‐type (WT) controls, and adoptive transfer of bone marrow‐derived mast cells (BMMCs) restored antifungal capacity, establishing a critical protective role for MCs in cutaneous *C. albicans* infection. Mechanistically, MCs secrete IL‐6 to drive antimicrobial peptide (AMP) production by keratinocytes (KCs). Reciprocally, KCs activate the non‐canonical NF‐κB (NF‐κB2) pathway and release CXCL2, which signals through CXCR2 on MCs to further promote IL‐6 secretion, establishing an NF‐κB2–CXCL2–IL‐6 amplification loop between the two cell types. Topical recombinant IL‐6 enhanced KC‐mediated antifungal activity and ameliorated cutaneous *C. albicans* infection in MC‐deficient mice, underscoring the essential, non‐redundant role of MC‐derived IL‐6 in this axis. Collectively, our findings reveal an MC‐KC amplification loop centered on the NF‐κB2–CXCL2–IL‐6 axis that orchestrates cutaneous antifungal defense and identify new therapeutic targets for cutaneous fungal infection.

## Graphical abstract

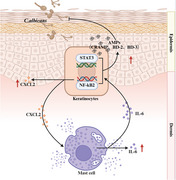

## 1 Introduction

*Candida albicans* (*C. albicans*) is a dimorphic fungus that exists as a commensal on healthy human skin, mucosa and the reproductive tract, but it is also one of the most prevalent opportunistic fungal pathogens, capable of causing diseases ranging from superficial cutaneous candidiasis to chronic mucocutaneous candidiasis and life‐threatening disseminated infections [1, 2, 3]. Of particular concern, the global incidence of candidiasis has risen substantially in recent decades, and over 10% of cases now exhibit resistance to antifungal agents [4]. These trends highlight an urgent need to elucidate the mechanisms of cutaneous infection and host antifungal defense. Despite advances in understanding systemic antifungal immunity, the regulatory networks coordinating local innate immune responses in *C. albicans*‐infected skin, in particular the cooperative mechanisms among different innate cell types, remain poorly defined.

Mast cells (MCs), long recognized for their pathogenic role in allergic disorders, are increasingly acknowledged as pivotal effectors of the innate immune system in host defense against pathogens [5, 6]. In bacterial infections, MCs release a broad repertoire of bioactive mediators, such as tumor necrosis factor‐α (TNF‐α), leukotrienes, and serine proteases, which together promote pathogen clearance and recruit neutrophils, dendritic cells, and T cells to sites of infection [7]. However, the functional role of MCs in *C. albicans i*nfection remains controversial. Several in vitro studies have suggested that MCs exert a protective effect against *C. albicans* by modulating antifungal immunity, whereas other investigations report that MCs form extracellular traps capable of physically restricting fungal dissemination without altering fungal viability [8]. Whereas the cooperation of MCs with other immune cells during bacterial infection has been extensively documented, their potential crosstalk with skin‐resident non‐immune cells (such as KCs that maintain cutaneous barrier function) during *C. albicans* infection has remained largely unexplored. This gap limits our understanding of how MCs integrate into the cutaneous antifungal defense network.

The cutaneous epithelium, primarily composed of keratinocytes (KCs), constitutes the first mechanical and immunological barrier against *C. albicans* invasion [9]. As an opportunistic pathogen, *C. albicans* coexists with epithelial cells under steady‐state conditions. When host immunity is compromised or the cutaneous barrier is breached, *C. albicans* hyphae invade KCs via induced endocytosis and active penetration, thereby activating signaling pathways including NF‐κB, MPK1, and c‐FOS to initiate a protective inflammatory response [10, 11, 12]. Although KCs are well established as central players in cutaneous antifungal immunity, the extent to which they collaborate with MCs, another abundant skin‐resident innate cell type, to regulate *C. albicans* clearance remains poorly understood.

In the present study, we investigated the role of MCs and their cooperation with KCs in cutaneous *C. albicans* infection. Using human candidiasis specimens, MC‐deficient mice, and adoptive MC transfer, we first established a protective role for MCs in cutaneous antifungal defense. To further explore the specific mechanism, we investigated the synergistic antifungal defense between MCs and KCs through various in vitro co‐culture models. These data enhance our understanding of how MCs are integrated into the skin's antifungal defense network.

## 2 Results

### 2.1 MCs Are Abundant in Skin After *C. albicans* Infection

**FIGURE 1:**
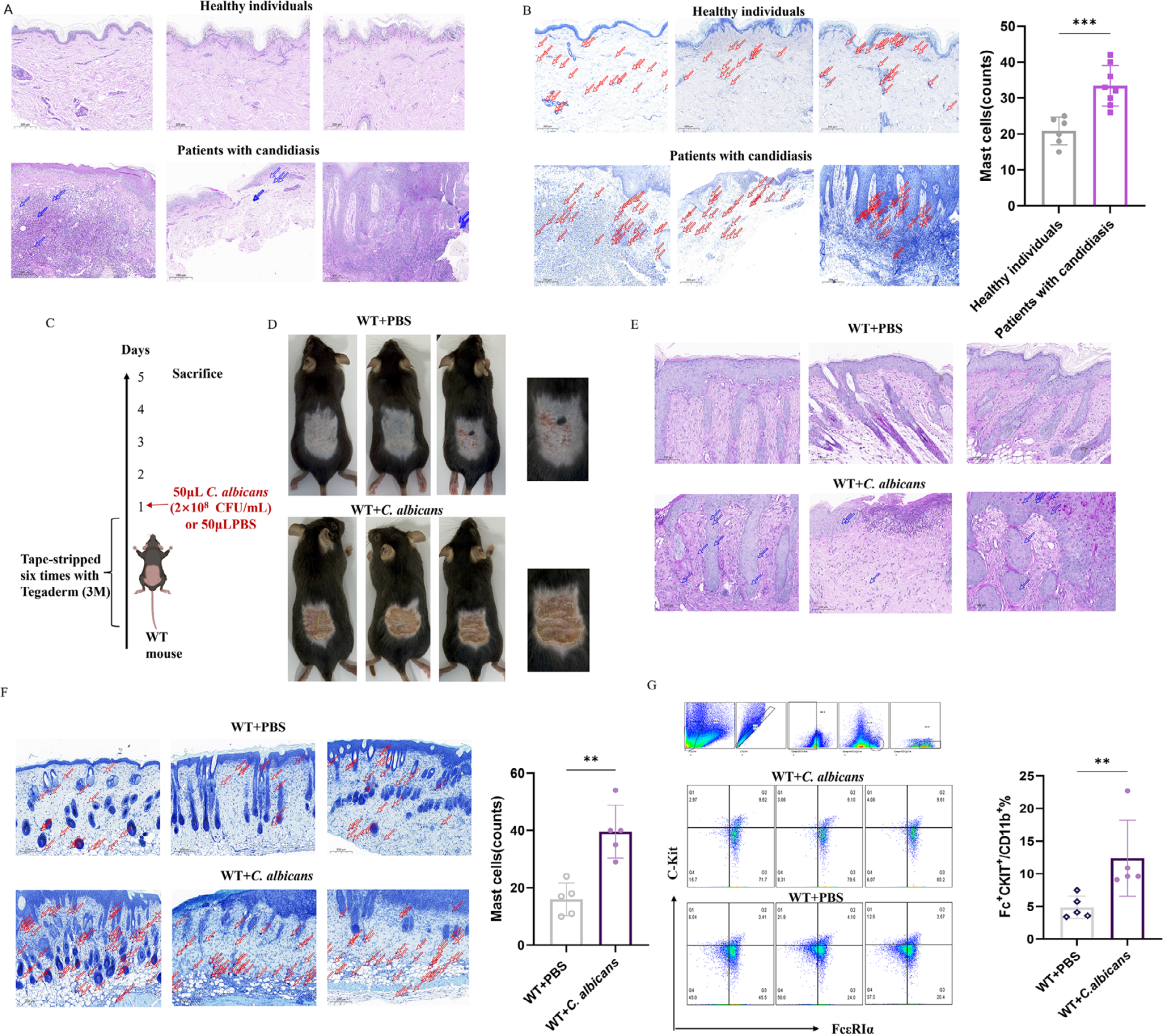
MCs are abundant in skin after *C. albicans* infection. (A). Representative images of PAS‐stained sections and fungal cultures from patients with candidiasis versus healthy controls. Fungal spores are indicated by blue arrows (Scale bar: 200 µm; n = 4). (B). Toluidine blue‐stained sections of skin lesions from candidiasis patients(n = 10) versus healthy controls(n = 6), with quantitative analysis of mast cell counts per field (Scale bar: 200 µm). Mast cells are indicated by red arrows. (C). Schematic diagram of the murine cutaneous *Candida* infection model. (D). Dorsal skin appearance of C57BL/6J mice infected with *C. albicans* versus PBS‐treated controls (n = 5). (E). Representative PAS‐stained sections and fungal cultures from WT+PBS versus WT+*C. albicans* groups (n = 5). (F). Toluidine blue‐stained skin sections with mast cell quantification in WT+PBS versus WT+*C. albicans* mouse (Scale bar: 200 µm; n = 5). Mast cells are indicated by red arrows. (G). Flow cytometric analysis of mast cell infiltration in skin lesions of WT+PBS versus WT+*C. albicans* mouse (n = 5). Data are presented as mean ± SD. *p < 0.05, **p < 0.01 and ***p < 0.001.

As tissue‐resident sentinels at barrier sites, MCs are among the earliest immune cells to encounter *C. albicans* during cutaneous infection. To examine whether MC numbers are altered in cutaneous candidiasis, we analyzed skin biopsies from patients with cutaneous candidiasis and from healthy controls. PAS staining confirmed the presence of fungal spores in patient lesions but not in control skin (Figure 1A). Toluidine blue staining revealed a significant increase in dermal MC counts in patient lesions compared with healthy controls (Figure 1B).

To recapitulate this observation in a controlled setting, we established a murine model of cutaneous *C. albicans* infection using C57BL/6J mice (Figure 1C). Infected mice developed marked erythema and scaling compared with PBS‐treated controls (Figure 1D), and PAS staining revealed abundant fungal spores together with sparse hyphae in infected skin (Figure 1E), consistent with previous reports [13]. Toluidine blue staining and flow cytometric analysis both showed a significant increase in dermal MC numbers in infected skin compared with PBS controls (Figure 1F, G). The above results confirmed that *C. albicans* infection significantly increased the number of MCs in the skin lesions.

### 2.2 MC Deficiency Aggravates Cutaneous *C. albicans* Infection in Mice

**FIGURE 2:**
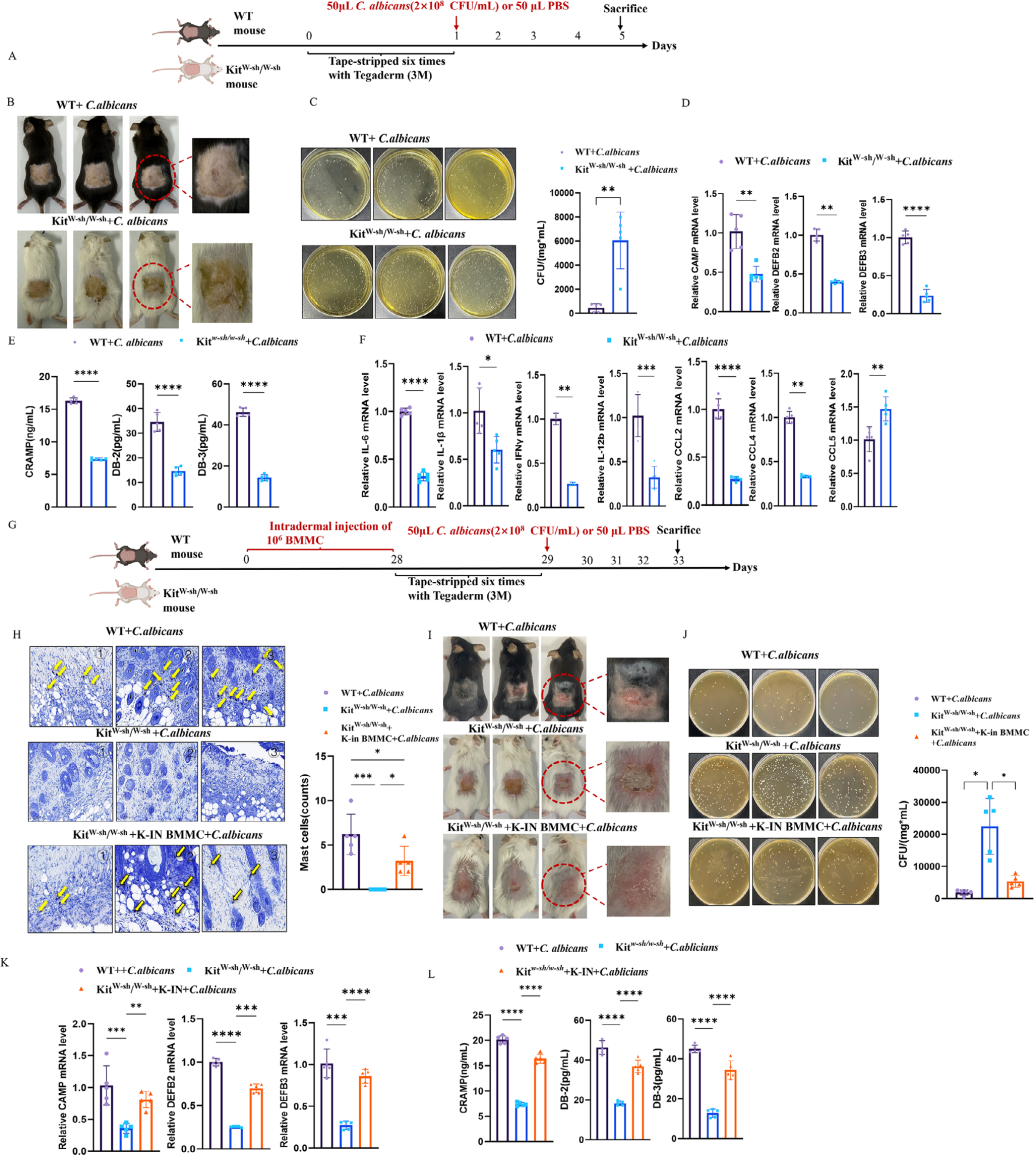
MC deficiency aggravates cutaneous *C. albicans* infection in mice. (A). Schematic diagram of the WT and Kit^W‐sh/W‐sh^ mouse cutaneous *C. albicans* infection model. (B). Representative dorsal skin images of WT and Kit^W‐sh/W‐sh^ mice after *C. albicans* infection (n = 5). (C) Representative CFU assays (left) and quantification (right) of *C. albicans* load in lesional skin homogenates (n = 5). (D). RNA was isolated from the skin of the two groups of mice, and analysis was performed to determine the indicated AMPs (CAMP, DEFB2 and DEFB3) mRNA expression (n = 5). (E). ELISA quantification of CRAMP, BD‐2, and BD‐3 protein levels in lesional skin homogenates from the two groups(n = 5). (F). qRT‐PCR analysis of inflammatory cytokines (IL‐6, IL‐1β, IFN‐γ, IL‐12b) and chemokines (CCL2, CCL4, CCL5) in lesional skin (n = 5). (G). Schematic of the BMMC adoptive transfer and cutaneous *C. albicans* infection model in Kit^W‐sh/W‐sh^ mice. (H). Representative toluidine blue staining of mast cells (yellow arrows, left) and quantification of dermal mast cell counts (right) in skin sections from WT, Kit^W‐sh/W‐sh^, and Kit^W‐sh/W‐sh^ + K‐in BMMC mice. (Scale bar: 100 µm; n = 5). (I). Representative dorsal skin images of the three groups after *C. albicans* infection (n = 5). (J). Representative CFU assays (left) and quantification (right) of *C. albicans* load in lesional skin homogenates from the three groups(n = 5). (K). qRT‐PCR analysis of AMPs (CAMP, DEFB2, DEFB3) mRNA expression in skin tissues from the three groups (n = 5). L. ELISA quantification of CRAMP, BD‐2, and BD‐3 levels in lesional skin homogenates from all three groups (n = 5). Data are presented as mean ± SD. *p < 0.05; **p < 0.01; ***p < 0.001; ****p < 0.0001.

To directly test the role of MCs in cutaneous *C. albicans* defense, we inoculated MC‐deficient Kit^W‐sh/W‐sh^ mice and WT mice with *C. albicans* (Figure 2A). Kit^W‐sh/W‐sh^ mice developed thicker scabs and carried higher fungal loads than WT controls (Figure 2B, C). At the molecular level, lesional skin of Kit^W‐sh/W‐sh^ mice showed markedly lower transcript levels (Figure 2D) and protein secretion (Figure 2E) of the antimicrobial peptides CAMP, BD‐2, and BD‐3. In parallel, inflammatory cytokines (IL‐6, IL‐1β, IFN‐γ, IL‐12b) and chemokines (CCL2, CCL4, CCL5) were substantially reduced in Kit^W‐sh/W‐sh^ mouse skin compared with WT skin (Figure 2F). Together, these results suggest that MC deficiency compromises cutaneous antifungal defense through diminished AMP production and a blunted local inflammatory response.

To test whether the aggravated phenotype of Kit^W‐sh/W‐sh^ mice was specifically caused by MC deficiency rather than other effects of the Kit mutation, we performed local adoptive transfer of cultured BMMCs (K‐in BMMC) into the dorsal skin of Kit^W‐sh/W‐sh^ recipients four weeks prior to infection (Figure 2G). Successful reconstitution of cutaneous MCs was confirmed by toluidine blue staining (Figure 2H). Reconstituted Kit^W‐sh/W‐sh^+K‐in BMMC mice exhibited less severe skin lesions and lower fungal burdens than unreconstituted Kit^W‐sh/W‐sh^ mice, with outcomes approaching those of WT controls (Figure 2I, J). Correspondingly, both transcript and protein levels of CAMP, BD‐2, and BD‐3 in lesional skin were largely restored following BMMC reconstitution (Figure 2K, L). These rescue data firmly establish that MCs are required for efficient antifungal defense in the skin.

### 2.3 MC‐KC Interaction Synergistically Resists *C. albicans*

**FIGURE 3:**
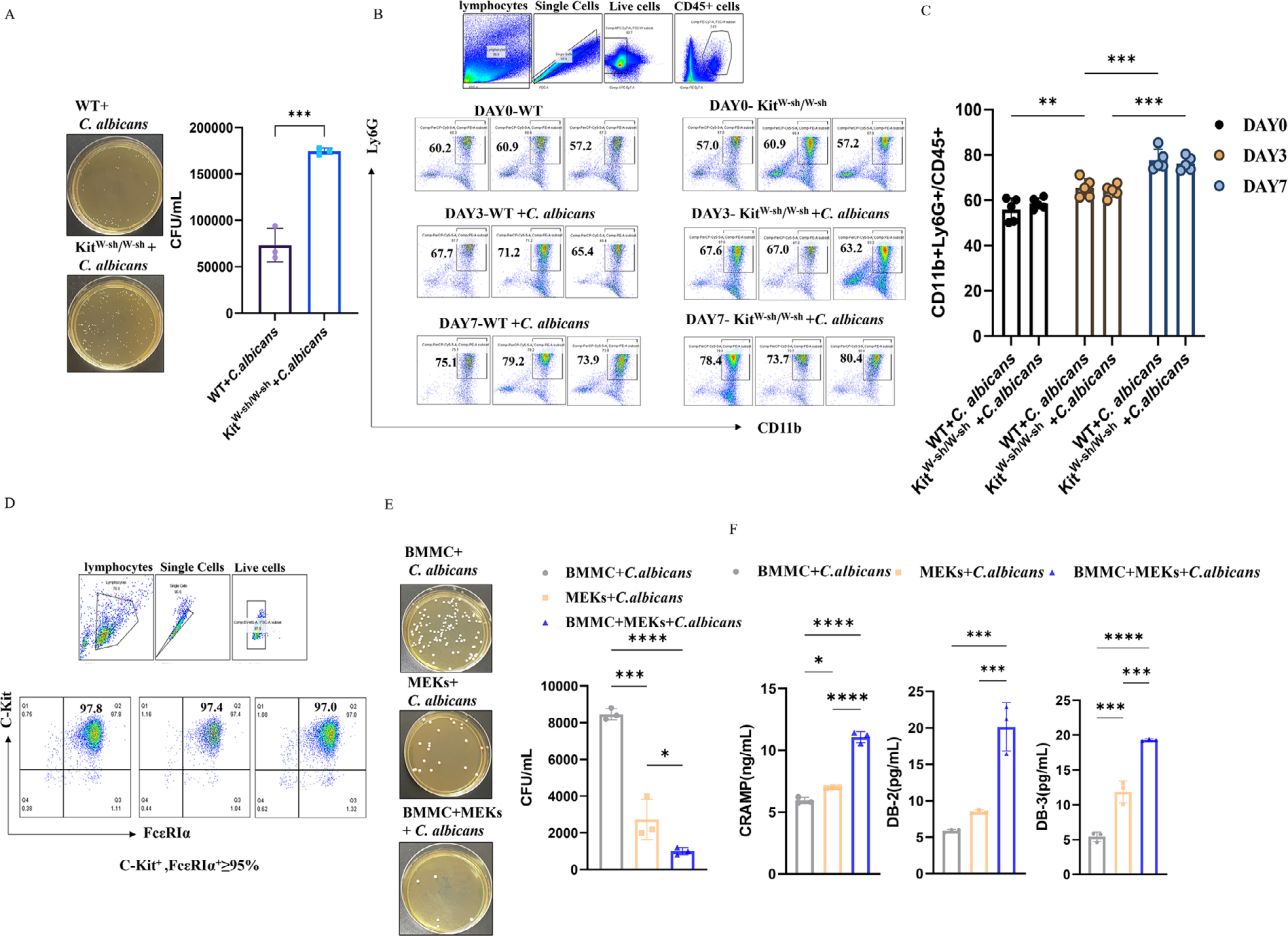
MC and KC synergistically restrict *C. albicans* growth in vitro. (A). Representative CFU images (left) and quantification (right) of *C. albicans* in ex vivo 5‐mm skin explants from WT and Kit^W‐sh/W‐sh^ mice infected with *C. albicans* (1 × 10^6^ CFU) for 24 h. (n = 3). (B, C). Flow cytometry analysis of neutrophil counts in skin lesions of Kit^W‐sh/W‐sh^ and WT mice at different time points (Day 0, 3, 7) post‐*C. albicans* infection (n = 5). (D). Flow cytometric purity assessment of isolated BMMCs (c‐Kit^+^FcεRIα^+^ ≥ 95%) (n = 3). (E). Representative CFU images (left) and quantification (right) of *C. albicans* under three co‐culture conditions: BMMCs + *C. albicans*, MEKs + *C. albicans*, and MEKs + BMMC+ *C. albicans*(n = 3). (F). ELISA measurement of AMPs (CRAMP, BD‐2, BD‐3) secretion levels in supernatants from the three co‐culture groups (n = 3). Data are presented as mean ± SD. *p < 0.05, **p < 0.01, ***p < 0.001, ****p < 0.0001.

We next asked whether the antifungal effect of MCs depends on the recruitment of circulating immune cells. To isolate the tissue‐resident immune compartment from circulating cells, we cultured 5‐mm ex vivo skin explants from Kit^W‐sh/W‐sh^ and WT mice in RPMI‐1640 medium with *C. albicans* (1 × 10^6^ CFU) for infection. Fungal loads were approximately two‐fold higher in Kit^W‐sh/W‐sh^ explants than in WT explants (Figure 3A), indicating that the MC‐dependent antifungal effect persists in the absence of circulating cell recruitment. To independently assess this, we quantified neutrophil infiltration by flow cytometry at multiple time points after infection and observed a comparable, time‐dependent increase in neutrophil numbers in both genotypes, with no significant difference between Kit^W‐sh/W‐sh^ and WT lesions (Figure 3B, C). Together, these data indicate that MCs contribute to cutaneous *C. albicans* clearance through a mechanism that does not rely on the recruitment of circulating innate immune cells.

To dissect the underlying mechanism, we moved to in vitro systems. BMMCs were generated from mouse bone marrow and confirmed to be ≥95% c‐Kit^+^FcεRIα^+^ by flow cytometry (Figure 3D). When co‐cultured with *C. albicans* alone (MOI = 1, 4 h), BMMCs did not significantly restrict fungal growth (Figure S1). Because KCs are known to secrete AMPs against *C. albicans* [14), we reasoned that MCs might act by cooperating with KCs rather than through direct antifungal activity. Consistent with this hypothesis, co‐culture of BMMCs with primary mouse epidermal keratinocytes (MEKs) at a 1:1 ratio significantly reduced fungal load compared with either monoculture (Figure 3E), and ELISA showed elevated CAMP, BD‐2, and BD‐3 secretion in BMMC‐MEK co‐cultures versus monocultures (Figure 3F). Thus, MCs and KCs act synergistically to enhance AMP‐dependent antifungal defense.

### 2.4 MC‐Derived IL‐6 Synergizes With KCs to Protect *C. albicans* Infection

**FIGURE 4:**
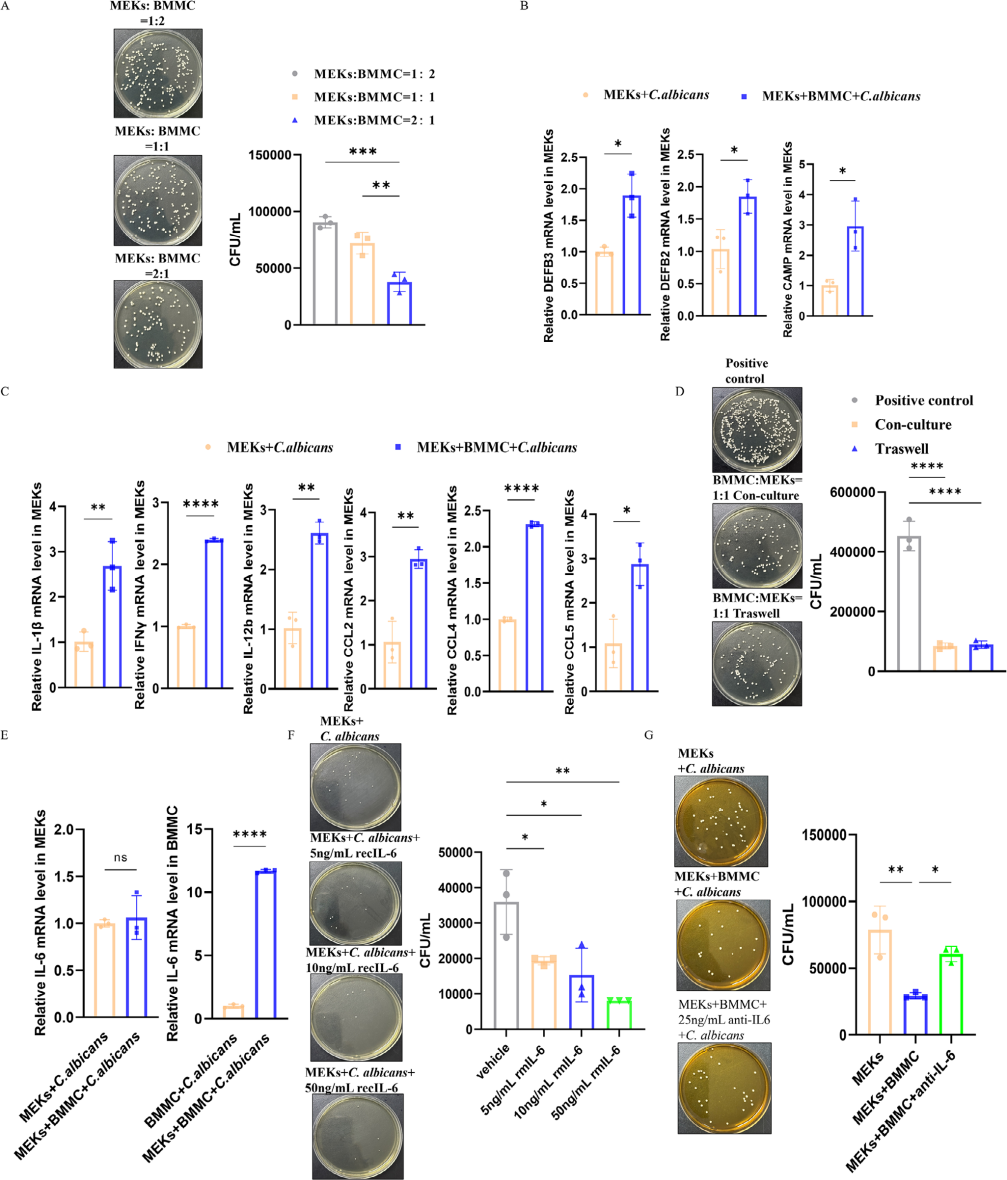
MC‐Derived IL‐6 synergizes with KCs to protect against *C. albicans* infection. (A). Quantification of *C. albicans* CFUs in co‐cultures with varying KC/MC ratios (MEKs: BMMC = 1:1, 2:1, and 1:2; n = 3). (B). qRT‐PCR analysis of DEFB3, DEFB2, and CAMP mRNA levels in MEKs from MEK+*C. albicans* and MEK+BMMC+*C. albicans* co‐cultures (MEK: BMMC = 1:1) (n = 3). (C). qRT‐PCR analysis of inflammatory cytokines (IL‐1β, IFN‐γ, IL‐12b) and chemokines (CCL2, CCL4, CCL5) in MEKs from two co‐cultures (n = 3). (D). Representative CFU images (left) and quantification (right) of C. albicans in direct‐contact or Transwell (0.4 µm pore) co‐cultures of MEKs and BMMCs(n = 3). (E). Total RNA was separately extracted from MEKs and BMMC in MEKs + BMMC + *C. albicans* co‐cultures (MEKs:BMMC = 1:1), and IL‐6 transcript levels were measured in both cell types (n = 3). (F). Representative CFU images (left) and quantification (right) of *C. albicans* in MEK+*C. albicans* cultures supplemented with rmIL‐6 at 0, 5, 10, or 50 ng/mL (n = 3). (G). Representative CFU images (left) and quantification (right) of *C. albicans* in MEK+BMMC+*C. albicans* co‐cultures with or without an anti‐IL‐6 neutralizing antibody (25 ng/mL) (n = 3). Data are presented as mean ± SD. *p < 0.05, **p < 0.01, ****p < 0.0001.

To further characterize this synergistic antifungal effect, we varied the MEK:BMMC ratio while keeping the total cell number constant. The antifungal activity increased with higher KC:MC ratios (Figure 4A), indicating that KCs are the principal effectors while MCs serve a potentiating role. The same pattern held in human cells: co‐culture of the human keratinocyte line HaCaT with the human mast cell line LAD2 recapitulated the synergistic reduction in fungal load (Figure S2A). At the transcriptional level, qRT‐PCR analysis of MEKs from MEK+*C. albicans* versus MEK+BMMC+*C. albicans* co‐cultures revealed that the presence of BMMCs significantly upregulated CAMP, DEFB2, and DEFB3 in MEKs (Figure 4B), together with a broader induction of inflammatory cytokines (IL‐1β, IFN‐γ, IL‐12b) and chemokines (CCL2, CCL4, CCL5) (Figure 4C).

To determine whether this synergy required direct cell‐cell contact, we physically separated MEKs and BMMCs using a Transwell insert during co‐culture. Transwell separation did not abolish the antifungal synergy (Figure 4D), indicating that the MC‐KC cooperation is mediated by soluble factors rather than direct contact.

To identify the soluble mediator responsible for this synergy, we profiled culture supernatants by multiplex secretome analysis and identified IL‐6 as one of the most strongly induced cytokines in MEK‐BMMC co‐cultures infected with *C. albicans* (Figure S3). To determine the cellular source of IL‐6, we measured Il6 transcript levels in the two cell types after sorting from infected co‐cultures. Il6 mRNA was approximately 11‐fold higher in BMMCs than in MEKs, identifying MCs as the primary producer of IL‐6 in this system (Figure 4E). Functionally, supplementing MEK‐*C. albicans* monocultures with recombinant mouse IL‐6 (rmIL‐6) reproduced the synergistic antifungal effect observed with BMMC co‐culture (Figure 4F), whereas addition of an IL‐6‐neutralizing antibody to MEK‐BMMC co‐cultures abolished it (Figure 4G). Importantly, IL‐6 production by BMMCs was observed only in the presence of MEKs (Figure 4E), suggesting that reciprocal KC‐derived signals are required to induce IL‐6 secretion by MCs.

### 2.5 MC‐Derived IL‐6 Promotes AMPs Production in KCs to Enhance Antifungal Effects

**FIGURE 5:**
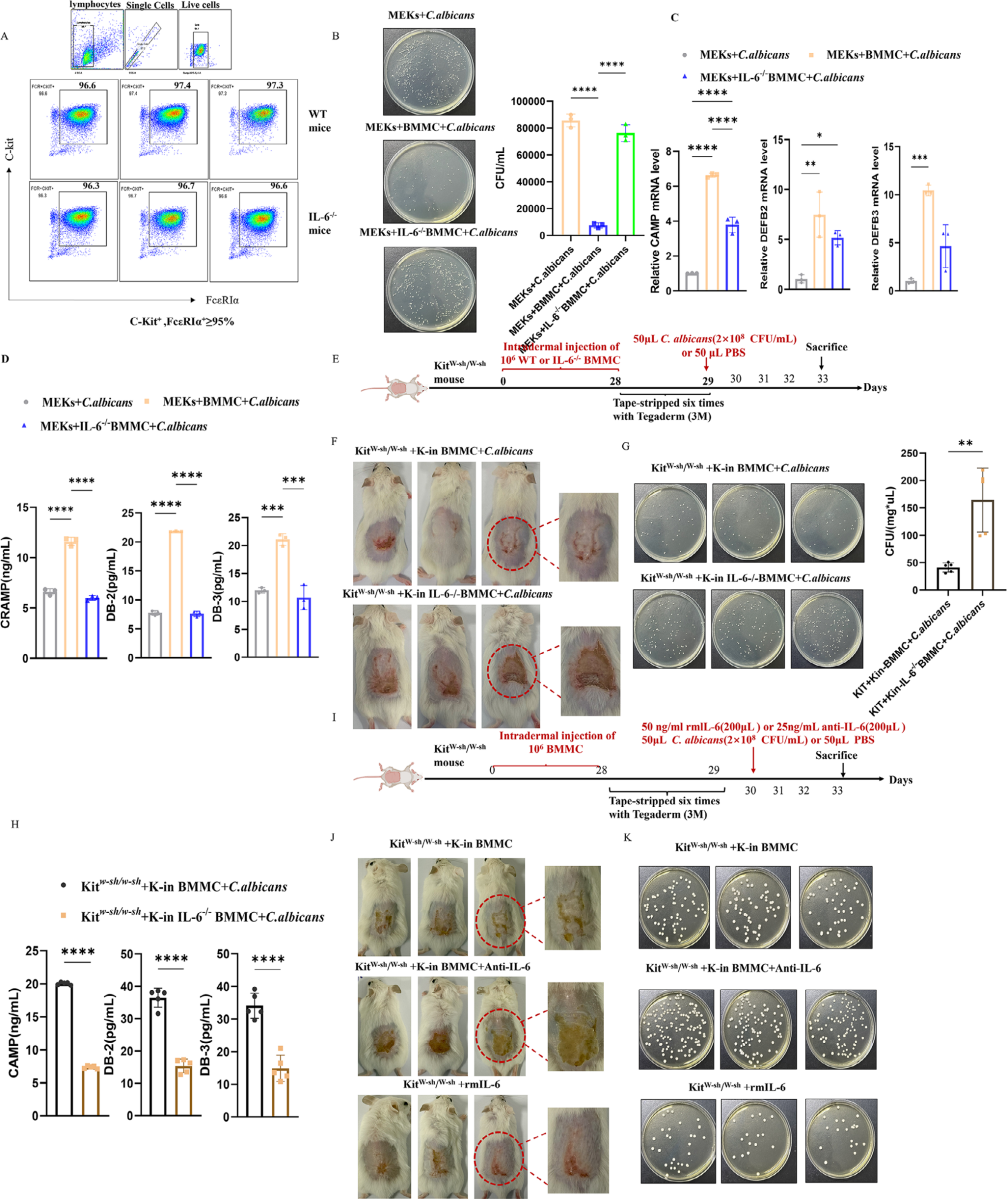
MC‐derived IL‐6 promotes AMP production in KCs to enhance antifungal effects. (A). Flow cytometric purity assessment of BMMCs derived from WT and IL‐6^−/−^ mice (c‐Kit^+^FcεRIα^+^≥ 95%, n = 3). (B). Representative CFU images (left) and quantification (right) of *C. albicans* in MEK+*C. albicans*, MEK+BMMC+*C. albicans*, or MEK+IL‐6^−/−^ BMMC+*C. albicans* co‐cultures (n = 3). (C). qRT‐PCR analysis of CAMP, DEFB2, and DEFB3 mRNA levels in MEKs from the three co‐culture conditions(n = 3). (D). ELISA measurement of the secretion levels of AMPs (CRAMP, BD‐2, BD‐3) in the supernatants from the three co‐culture groups (n = 3). (E). Schematic of the in vivo adoptive transfer experiment: Kit^W‐sh/W‐sh^ mice received intradermal transfer of WT BMMCs (Kit^W‐sh/W‐sh^ + K‐in BMMC) or IL‐6^−/−^ BMMCs (Kit^W‐sh/W‐sh^ + K‐in IL‐6^−/−^ BMMC) four weeks prior to cutaneous *C. albicans* challenge. (F). Representative dorsal skin images of the two groups after *C. albicans* infection (n = 5). (G). Representative CFU images (left) and quantification (right) of *C. albicans* in lesional skin homogenates from the two groups (n = 5). (H). ELISA quantification of CRAMP, BD‐2, and BD‐3 levels in lesional skin homogenates from the Kit^W‐sh/W‐sh^ + K‐in BMMC group and the Kit^W‐sh/W‐sh^ + K‐in IL‐6^−/−^ BMMC group (n = 5). (I). Schematic diagram of the procedure for establishing the skin candidiasis model in Kit^W‐sh/W‐sh^ mice, divided into three groups: Kit^W‐sh/W‐sh^ + K‐in BMMC group, Kit^W‐sh/W‐sh^ + K‐in BMMC + Anti‐IL‐6 group, and Kit^W‐sh/W‐sh^ + rmIL‐6 group. (J). Representative dorsal skin images of the three groups after *C. albicans* infection (n = 5). (K). Representative CFU images (left) and quantification (right) of *C. albicans* in lesional skin homogenates from the three groups (n = 5). Data are presented as mean ± SD. *p < 0.05, **p < 0.01, ***p < 0.001, ****p < 0.0001.

To directly test the requirement for MC‐derived IL‐6, we generated BMMCs from IL‐6^−/−^ and WT mice and confirmed ≥95% purity (c‐Kit^+^FcεRIα^+^) in both genotypes (Figure 5A). IL‐6^−/−^ BMMCs failed to cooperate with MEKs to restrict *C. albicans* growth in co‐culture (Figure 5B). Correspondingly, MEKs co‐cultured with IL‐6^−/−^ BMMCs showed significantly lower CAMP, DEFB2, and DEFB3 transcript levels and markedly reduced CRAMP, BD‐2, and BD‐3 protein secretion compared with MEKs co‐cultured with WT BMMCs (Figure 5C, D). These data establish that MC‐derived IL‐6 is required for AMP induction in KCs during *C. albicans* infection.

To assess the in vivo relevance of this axis, we performed local adoptive transfer of WT or IL‐6^−/−^ BMMCs into Kit^W‐sh/W‐sh^ mice prior to *C. albicans* challenge (Figure 5E). Recipients reconstituted with IL‐6^−/−^ BMMCs developed more severe skin lesions (Figure 5F) and carried significantly higher fungal loads (Figure 5G) than those reconstituted with WT BMMCs. Consistent with the in vitro findings, lesional CRAMP, BD‐2, and BD‐3 protein levels were markedly reduced in mice receiving IL‐6^−/−^ BMMCs compared with WT BMMC recipients (Figure 5H). These in vivo data confirm that MC‐derived IL‐6 is required for optimal cutaneous antifungal defense.

We next asked whether exogenous IL‐6 could rescue the MC‐deficient phenotype, and whether blocking IL‐6 could override the protection provided by BMMC reconstitution. Kit^W‐sh/W‐sh^ mice received local pretreatment with rmIL‐6, anti‐IL‐6, or vehicle prior to *C. albicans* challenge (Figure 5I). rmIL‐6 pretreatment significantly improved antifungal outcomes in Kit^W‐sh/W‐sh^ mice, whereas anti‐IL‐6 administration in BMMC‐reconstituted Kit^W‐sh/W‐sh^ mice nullified the rescue and aggravated cutaneous infection, with fungal loads exceeding those in unreconstituted Kit^W‐sh/W‐sh^ controls (Figure 5J, K). To extend these findings to the human system, we performed analogous in vitro infection experiments using the human keratinocyte line HaCaT: recombinant human soluble IL‐6(rhIL‐6) pretreatment significantly enhanced *C. albicans* clearance compared with vehicle (FigureS2B), indicating that the IL‐6‐dependent antifungal mechanism is conserved in human KCs.

### 2.6 CXCL2 Promotes IL‐6 Transcription and Secretion in MCs via the CXCR2 Receptor

Thus far, our data demonstrate that MC‐derived IL‐6 drives AMP production by KCs and enhances antifungal defense. We next asked whether KCs reciprocally signal back to MCs to promote IL‐6 secretion, thereby constituting a bidirectional amplification loop.

**FIGURE 6:**
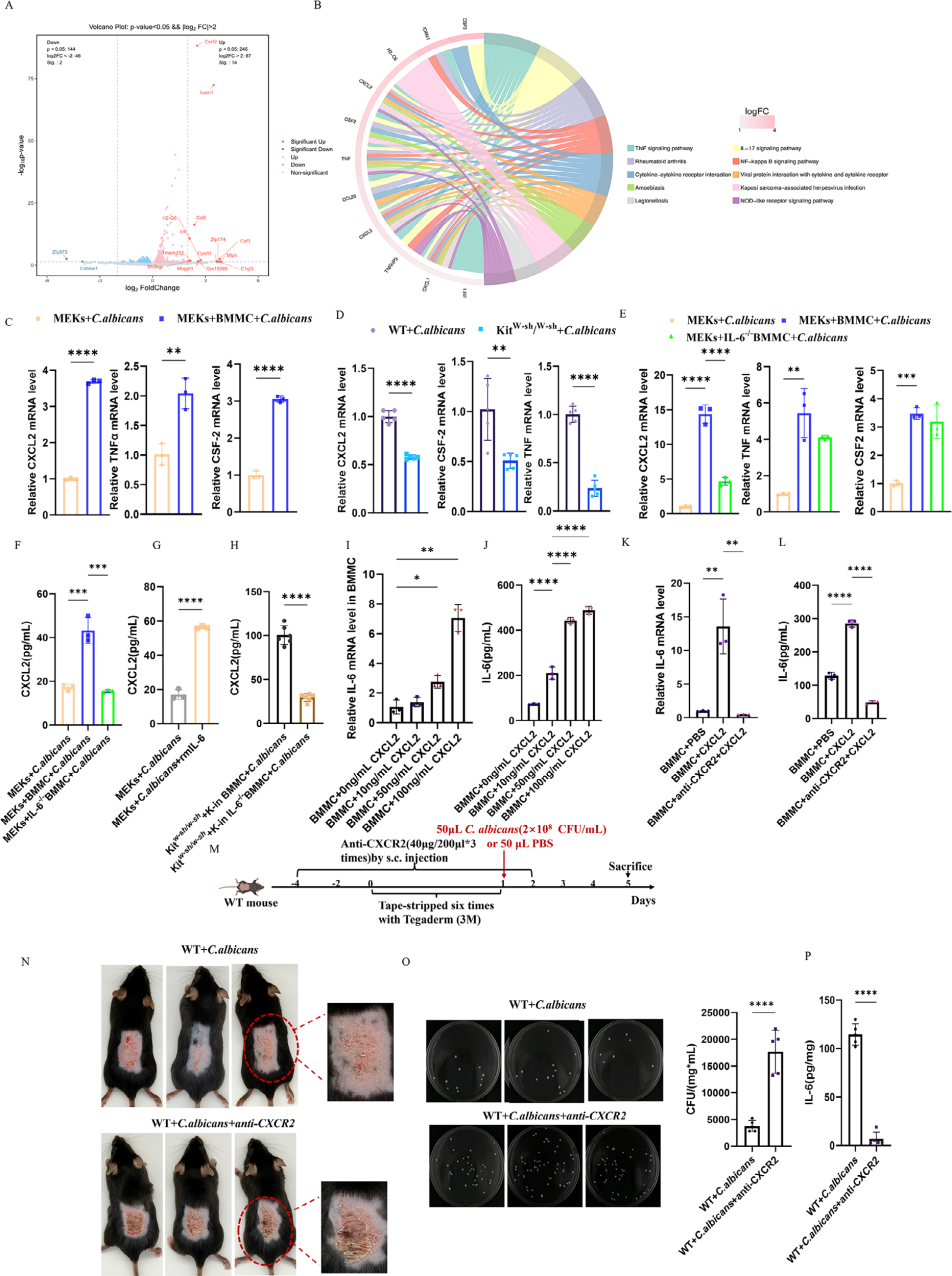
KCs induce IL‐6 secretion in MCs via the CXCL2‐CXCR2 axis. (A). Volcano plot of differentially expressed genes in KCs between the MEKs + *C. albicans* and MEKs + BMMC + *C. albicans* groups (*P*< 0.05 and |logFC| > 2) (n = 3).​ (B). Chord diagram of KEGG enrichment analysis(n = 3).​ (C). qRT‐PCR analysis of CXCL2, TNF‐α, and CSF‐2 mRNA levels in MEKs from MEK+*C. albicans* and MEK+BMMC+*C. albicans* co‐cultures (n = 3).​ (D). qRT‐PCR of analysis CXCL2, TNF‐α, and CSF‐2 mRNA levels in lesional skin from WT and Kit^W‐sh/W‐sh^ mice after *C. albicans* infection (n = 5).​ (E). qRT‐PCR analysis of CXCL2, TNF‐α, and CSF‐2 mRNA levels in MEKs from MEK+*C. albicans*, MEK+BMMC+*C. albicans*, and MEK+IL‐6^−/−^ BMMC+*C. albicans* co‐cultures (n = 3). (F). ELISA measurement of CXCL2 levels in different co‐culture systems (n = 3).​ (G). ELISA quantification of CXCL2 in supernatants from MEKs stimulated with or without rmIL‐6 in the presence of *C. albicans* (n = 3).​ (H). ELISA quantification of CXCL2 in lesional skin homogenates from Kit^W‐sh/W‐sh^ mice reconstituted with WT BMMCs or IL‐6^−/−^ BMMCs after *C. albicans* infection (n = 5).​ (I). qRT‐PCR analysis of the effect of different concentrations of CXCL2 (10 ng/mL, 50 ng/mL, and 100 ng/mL) on IL‐6 transcriptional levels in BMMC (n = 3).​ (J). ELISA detection of the effect of different concentrations of CXCL2 on IL‐6 secretion in BMMC (n = 3).​ (K). qRT‐PCR of analysis of the effect of CXCL2 or anti‐CXCR2 on IL‐6 secretion in BMMC (n = 3). (L). ELISA detection of the effect of CXCL2 or anti‐CXCR2 on IL‐6 secretion in BMMC (n = 3).​ (M). Schematic of the in vivo anti‐CXCR2 neutralization experiment: WT mice received subcutaneous injection of anti‐CXCR2 antibody (40 µg in 200 µL, three doses) prior to cutaneous *C. albicans* infection. (N). Representative macroscopic images of skin lesions in the control group and the anti‐CXCR2 treated group (n = 5). (O). Representative CFU images from lesional skin of the two groups(n = 5). Fungal burden quantification (CFU/g) in the skin tissues of two groups (n = 5). P. ELISA detection of IL‐6 protein secretion levels in the skin lesion tissues (n = 5).​ Data are presented as mean ± SD. *p < 0.05; **P < 0.01; ***p < 0.001; ****P < 0.0001.

To address this, we performed RNA‐seq on sorted KCs from MEK+*C. albicans* versus MEK+BMMC+*C. albicans* co‐cultures. Using a threshold of p < 0.05 and |log∼2∼FC| > 2, we identified 14 upregulated genes in KCs from the co‐culture condition (Figure 6A). KEGG enrichment analysis followed by intersection of the top differentially expressed genes across enriched pathways (chord diagram, Figure 6B) revealed six common candidates: CSF3, CXCL2, ICAM1, CSF2, TNF, and H2‐Q6. Among these, CXCL2, CSF2, and TNF have been reported to trigger IL‐6 release from MCs [15, 16, 17]. qRT‐PCR confirmed significantly elevated transcript levels of CXCL2, CSF2, and TNF‐α in MEKs from MEK+BMMC+*C. albicans* co‐cultures (Figure 6C), and conversely, these transcripts were markedly reduced in lesional skin of Kit^W‐sh/W‐sh^ mice compared with WT littermates after *C. albicans* infection (Figure 6D).

Interestingly, co‐culture of MEKs with IL‐6^−/−^ BMMCs failed to induce the upregulation of CXCL2 observed with WT BMMCs (Figure 6E), suggesting that MC‐derived IL‐6 is needed for KC‐derived CXCL2 production. Consistent with this, CXCL2 protein secretion was significantly lower in MEK+ IL‐6^−/−^ BMMC+*C. albicans* co‐cultures than in WT BMMC co‐cultures (Figure 6F), and this defect was restored by supplementation with rmIL‐6 (Figure 6G). In vivo, lesional CXCL2 protein levels were markedly lower in Kit^W‐sh/W‐sh^ mice reconstituted with IL‐6^−/−^ BMMCs than in those reconstituted with WT BMMCs (Figure 6H). Together, these results place MC‐derived IL‐6 upstream of KC‐derived CXCL2 production.

We next examined whether CXCL2 directly stimulates IL‐6 secretion by MCs and, if so, through which receptor. Stimulation of BMMCs with recombinant CXCL2 produced a dose‐dependent increase in both IL‐6 transcript and IL‐6 protein levels (Figure 6I, J). Pretreatment of BMMCs with an anti‐CXCR2 neutralizing antibody completely abrogated the CXCL2‐induced IL‐6 response (Figure 6K, L), establishing CXCR2 as the cognate receptor on MCs.

To validate the in vivo relevance of this axis, we administered an anti‐CXCR2 neutralizing antibody locally to WT mice prior to *C. albican*s infection (Figure 6M). CXCR2 blockade markedly aggravated skin lesions (Figure 6N), increased fungal burden (Figure 6O), and significantly reduced lesional IL‐6 secretion (Figure 6P). Together, these data demonstrate that KC‐derived CXCL2 activates MCs to release IL‐6 through CXCR2, and that this axis is critical for cutaneous antifungal defense in vivo.

To gain a broader view of the MC transcriptional response to CXCL2, we performed RNA‐seq on BMMCs treated with *C. albicans* alone or *C. albicans* + CXCL2. A total of 275 differentially expressed genes (230 upregulated, 45 downregulated; q < 0.05, |log∼2∼FC|>1) were identified (Figure S4A). CXCL2 stimulation upregulated inflammation‐associated genes (FCNB, SLC28A2) and downregulated selected metabolic and transcriptional regulators (MMP13, ZFP811, CEACAM10). KEGG enrichment highlighted the phagosome pathway and the interleukin‐17 (IL‐17) signaling pathway as the most prominent pathways modulated (Figure S4B). These data indicate that CXCL2‐CXCR2 signaling in MCs coordinates a broader inflammatory program extending beyond IL‐6 induction.

### 2.7 CXCL2‐mediated IL‐6 Secretion Drives AMP Production in KC Through NF‐κB2 and STAT3 Activation

**FIGURE 7:**
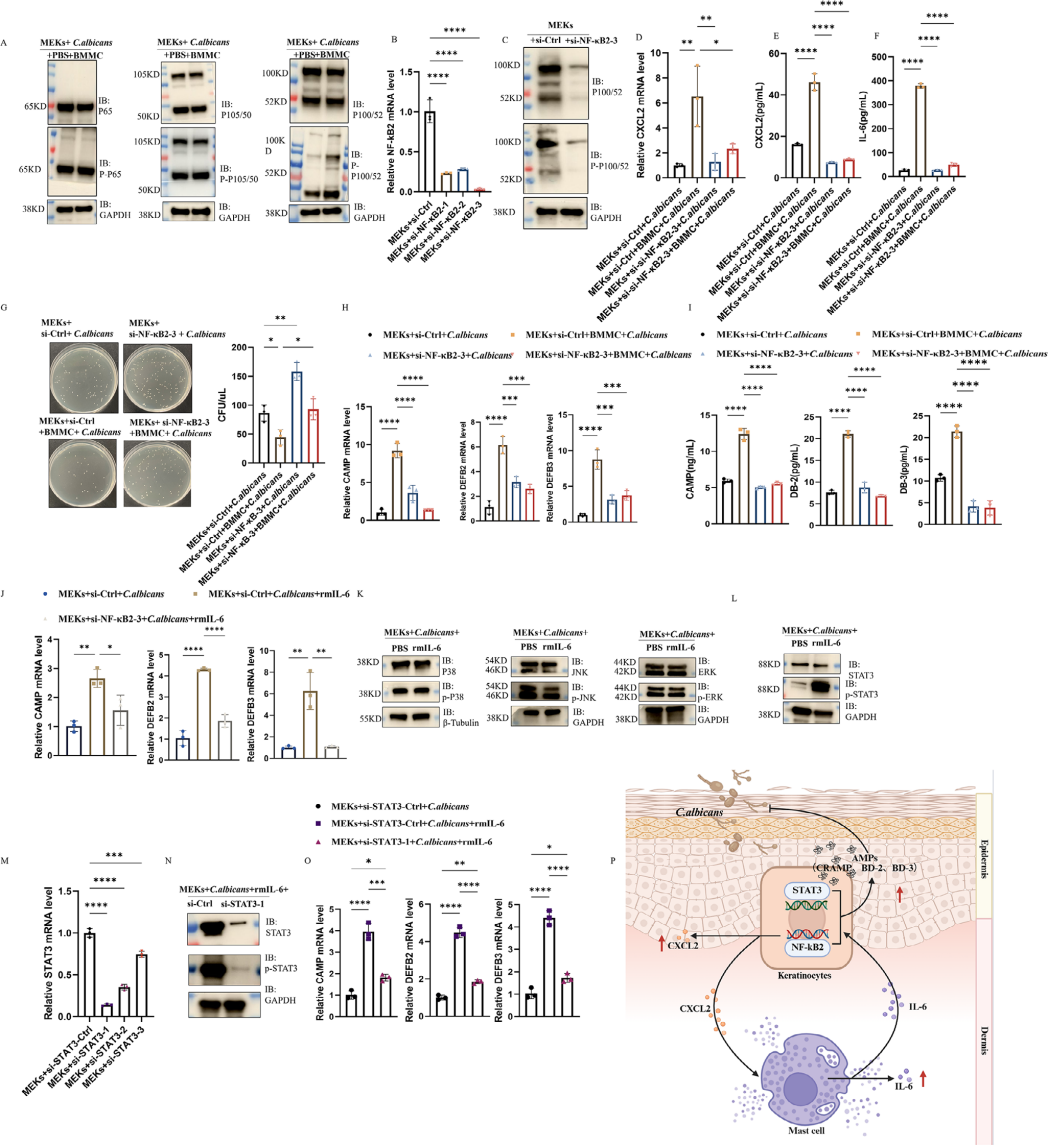
CXCL2‐mediated IL‐6 secretion drives AMP production in KC through NF‐κB2 and STAT3 activation. (A). Western blot (WB) analysis of the activation status of the NF‐κB pathway (P65, P100/52, P105/50) in KCs. (B). qRT‐PCR analysis of NF‐κB2 mRNA levels in MEKs after transfection with control siRNA (si‐Ctrl) or three independent NF‐κB2 siRNAs (si‐1, si‐2, si‐3) (n = 3). (C). Western blot analysis of p100/p52 and p‐p100/p52 protein levels in MEKs after si‐NF‐κB2‐3 interference. (D). qRT‐PCR analysis of CXCL2 mRNA levels in MEKs from four conditions: si‐Ctrl+*C. albicans*, si‐Ctrl+BMMC+*C. albicans*, si‐NF‐κB2‐3+*C. albicans*, and si‐NF‐κB2‐3+BMMC+*C. albicans* (n = 3). (E). ELISA measurement of CXCL2 secretion levels in the co‐culture system after siRNA interference (n = 3). (F). ELISA detection of IL‐6 secretion levels in the co‐culture system after siRNA interference (n = 3). (G). Representative CFU images (left) and quantification (right) of *C. albicans* in co‐culture supernatants from the four conditions (n = 3). (H). qRT‐PCR analysis of AMPs (CAMP, DEFB2, DEFB3) transcriptional levels in MEKs after siRNA interference (n = 3). (I). ELISA measurement of AMPs (CRAMP, BD‐2, BD‐3) secretion levels in the co‐culture system after siRNA interference (n = 3). (J). qRT‐PCR analysis of CAMP, DEFB2, and DEFB3 mRNA levels in MEKs pretreated with si‐Ctrl or si‐NF‐κB2‐3, followed by rmIL‐6 stimulation in the presence of *C. albicans* (n = 3). (K, L). WB analysis of signaling pathways in MEKs after rmIL‐6 stimulation, including phosphorylated and total p38, JNK, ERK and STAT3. (M). qRT‐PCR analysis of STAT3 mRNA levels in MEKs after transfection with control siRNA or three independent STAT3 siRNAs (si‐STAT3‐1, ‐2, ‐3) (n = 3). (N). WB detection of STAT3 and p‐STAT3 protein levels after si‐STAT3‐1 interference in MEKs stimulated with rmIL‐6. (O). qRT‐PCR analysis of AMPs (CAMP, DEFB2, DEFB3) mRNA levels in MEKs after si‐STAT3 interference under rmIL‐6 stimulation (n = 3). P. Schematic diagram of the proposed mechanism: *C. albicans* infection triggers the NF‐κB2‐CXCL2 axis in KCs, inducing MCs to secrete IL‐6. IL‐6 then acts back on KCs to promote AMP production (CRAMP, BD‐2, BD‐3) via NF‐κB2 and STAT3 signaling, facilitating fungal clearance. Data are presented as mean ± SD. *p < 0.05, **p < 0.01, ***p < 0.001, ****p < 0.0001.

Previous studies have reported that the production of CXCL2 is significantly related to the activation of NF‐κB signaling [18, 19]. KEGG enrichment analysis of our earlier RNA‐seq data pointed to activation of the NF‐κB signaling pathway in KCs during co‐culture with BMMCs (Figure S5). Because the NF‐κB family comprises both canonical (p65, p105/p50) and non‐canonical (p100/p52, also known as NF‐κB2) branches, we next determined which subunit is responsible for CXCL2 induction in KCs. Western blot analysis of MEKs from MEK+*C. albicans* versus MEK+BMMC+*C. albicans* co‐cultures revealed no marked changes in p‐p65 or p‐p105/p50, whereas p‐p100/p52 (indicative of NF‐κB2 activation) was selectively and strongly upregulated in the co‐culture condition (Figure 7A).

To functionally confirm the role of NF‐κB2, we designed three independent NF‐κB2 siRNAs; qRT‐PCR and Western blot confirmed that si‐ NF‐κB2‐3 most effectively reduced both NF‐κB2 mRNA and p100/p52 protein levels (Figure 7B, C). In MEKs transfected with si‐NF‐κB2‐3, CXCL2 transcript and CXCL2 protein were significantly reduced in the co‐culture system (Figure 7D, E), together with a concomitant decrease in IL‐6 secretion (Figure 7F), a higher fungal load (Figure 7G), and attenuated AMP induction both in transcript and protein levels (Figure 7H, I). Thus, NF‐κB2 activation in KCs is required for CXCL2‐driven engagement of MCs and for AMP induction.

Interestingly, we found that knocking down NF‐κB2 in KCs partially decreased the rmIL‐6‐induced expression of AMPs (Figure 7J), suggesting a coordinated regulatory network. To elucidate the primary specific intracellular signaling cascades downstream of IL‐6 that orchestrate AMP production in KCs, we systematically interrogated classical IL‐6 downstream pathways, including MAPK and STAT3. WB analysis revealed no significant differences in the total or phosphorylated levels of MAPK pathway proteins (P38, JNK, ERK) in MEKs following rmIL‐6 stimulation (Figure 7K), indicating that IL‐6‐mediated AMP secretion is largely independent of MAPK activation. Conversely, WB analysis demonstrated a robust activation of STAT3 phosphorylation (p‐STAT3) in KCs upon rmIL‐6 stimulation (Figure 7L). To definitively prove that STAT3 drives AMP production, we utilized a highly effective siRNA targeting STAT3 (si‐STAT3‐1) (Figure 7M, N). Crucially, knocking down STAT3 in KCs significantly abrogated the rmIL‐6‐induced upregulation of AMPs (CAMP, DEFB2, DEFB3) (Figure 7O).

To evaluate the clinical relevance of this amplification loop, we expanded our cohort and stratified patients into mild and severe infection groups based on the composite H&E histopathological score (see Methods). TSA‐based immunofluorescence staining of lesional skin sections revealed that CXCL2 expression was significantly elevated in both patient groups compared with healthy controls, with the highest levels in the severe group (Figure S6A, B). IL‐6 expression showed a similar but distinct pattern: compared with healthy controls, IL‐6 was not significantly altered in mild cases but was markedly upregulated in severe cases (Figure S6C). These findings indicate that that CXCL2 and IL‐6 are actively involved in the pathogenesis of *C. albican*s skin infection.

Collectively, these data define a reciprocal MC‐KC amplification loop in which KC‐derived CXCL2, produced via NF‐κB2 activation, engages CXCR2 on MCs to drive IL‐6 secretion, and MC‐derived IL‐6 in turn acts through STAT3 in KCs to induce AMP production and enforce cutaneous antifungal defense (Figure 7P).

## 3 Discussion

MCs as innate immune cells resident in barrier tissues such as the skin and mucous membranes, have been widely recognized for their central role in anti‐infective defense [20, 21]. Their precise role in cutaneous *C. albicans* infection, however, has remained incompletely understood. Here we demonstrate that MCs are essential for cutaneous clearance of *C. albicans* by secreting IL‐6 to drive AMP production in KCs, and we identify a reciprocal NF‐κB2‐CXCL2‐IL‐6 amplification loop between KCs and MCs that sustains this defense.

Using MC‐deficient mice and co‐culture models, we showed that MC deficiency impairs fungal clearance and reduces the expression of key AMPs, including CRAMP, BD‐2, and BD‐3 [22, 23, 24]. These AMPs are known to disrupt *C. albicans* cell membranes and inhibit biofilm formation, underscoring their critical role at barrier sites. Previous studies have shown that KC‐MC crosstalk exerts a bidirectional regulatory role in several inflammatory disease [25, 26]. For instance, in the pathogenesis of psoriasis, KCs and MCs cooperate with each other to jointly drive the release of inflammatory factors and exacerbate psoriatic lesions [27]. We extend this paradigm to antifungal immunity, identifying MC‐derived IL‐6 to induce AMP production and enhance antifungal activity in murine and human KCs in vitro and in vivo (Figure 5). This finding provides a mechanistic basis for the increased risk of cutaneous infection in patients treated with the IL‐6 receptor antagonist tocilizumab, supporting a non‐redundant role of IL‐6 in cutaneous antifungal defense [28].

Our data also reframe the CXCL2‐CXCR2 axis, which has been studied primarily as a neutrophil‐homing chemokine signal [29, 30], as a bidirectional communication bridge between epithelial and tissue‐resident immune cells. While previous studies have suggested potential links between epithelial chemokines and immune activation [31, 32], we demonstrate that KCs drive IL‐6 secretion in MCs through the CXCL2‐CXCR2 axis. Both our in vitro neutralizing assays and in vivo anti‐CXCR2 administration models proved that blocking the CXCR2 receptor abolishes IL‐6 release and precipitates a severe infection phenotype, remarkably phenocopying the vulnerability of MC‐deficient mice. Importantly, MC‐dependent antifungal defense operated independently of neutrophil recruitment (Figure 3B, C), pointing to a cell‐type‐specific regulatory pattern grounded in direct epithelial‐MC crosstalk rather than classical leukocyte chemotaxis.

Downstream of this receptor‐ligand interaction, we identified that KCs produce CXCL2 via the activation of the NF‐κB2 (p‐P100/52) pathway. In reciprocation, the MC‐derived IL‐6 signals back to the KCs predominantly via NF‐κB2 (p‐P100/52) pathway and the classical STAT3 pathway, rather than the MAPK cascade, to drive the massive production of AMPs (CRAMP, BD‐2, BD‐3). The abrogation of AMP upregulation following STAT3 genetic knockdown firmly establishes STAT3 as the indispensable executing transcription factor in this cutaneous antifungal defense network [33].

Although this study delineates a synergistic immune map between MCs and KCs, future research should explore the initial recognition mechanisms, such as how MCs detect specific *C. albicans* PAMPs (e.g., β‐glucan), and whether other resident cells like macrophages or dendritic cells modulate this amplification loop. Regarding methodological choices, although qRT‐PCR transcripts were normalized to a single reference gene (GAPDH), the consistent convergence of mRNA data with orthogonal ELISA protein measurements across all key endpoints ensures the robustness of our conclusions. Nevertheless, a formal multi‐reference‐gene stability analysis remains a valuable direction for future refinemen. Furthermore, while longitudinal tracking of infection prognosis was constrained by clinical outpatient heterogeneity and diagnostic lags, our data establish that the CXCL2/IL‐6 axis correlates strongly with histopathological severity. Given that clinical severity dictates tissue damage and therapeutic difficulty, these findings underscore the translational significance of this signaling circuit in human candidiasis.

In conclusion, our study clarifies that MCs regulate cutaneous defense against *C. albicans* infection through the IL‐6‐STAT3‐AMPs axis and the KC‐NF‐κB2/CXCL2‐CXCR2 positive feedback loop (Figure 7P). This not only expands our understanding of the immune functions of MCs but also provides experimental evidence for the development of antifungal strategies targeting innate immunity. Future studies can focus on the clinical translation of this pathway to provide new approaches for the prevention and treatment of cutaneous fungal infections in immunocompromised populations.

## 4 Materials and Methods

### 4.1 Ethics Statement

The collection of skin tissue samples from all individuals has been approved by the Medical Ethics Committee of the Institute of Dermatology, Chinese Academy of Medical Sciences (Approval No: 2017‐KY‐022). All animal experiments involved in this study have been approved by the Experimental Animal Ethics Committee of the Institute of Dermatology, Chinese Academy of Medical Sciences (Approval No: 2024‐DW‐026).

### 4.2 Human Specimens

All clinical data and skin specimens from patients with cutaneous candidiasis and healthy controls were obtained with written informed consent from each participant, in accordance with the Declaration of Helsinki. An initial cohort of 10 patients and 6 healthy controls (Table S1) was used for histopathological analysis (Figure 1B). Subsequently, the patient cohort was expanded to 20 cases (Table S2), and stratified analysis was performed according to the severity of infection (Figure S6).

Inclusion criteria for patients were: (i) a clinical diagnosis of cutaneous infection; (ii)histopathological evidence of skin tissue infection as confirmed by a dermatopathologist; (iii) a positive fungal culture yielding *C. albicans* as the sole organism. Exclusion criteria were: (i) systemic immunocompromising conditions, such as HIV infection or hematologic malignancies; and (ii) mixed bacterial or non‐candidal fungal co‐infection at the biopsy site. Healthy control samples were obtained from the normal skin areas of patients undergoing blepharoplasty or epidermal grafting at our hospital; these individuals were confirmed to be free of any inflammatory or infectious diseases. For histological processing, all skin biopsy specimens were immediately fixed in 10% neutral buffered formalin for 6 to 24 h. Following fixation, tissues were trimmed, dehydrated through a graded ethanol series, cleared in xylene, and infiltrated with paraffin. The specimens were then embedded in paraffin blocks with the epidermis oriented upward and allowed to solidify for subsequent sectioning.

### 4.3 *C. albicans* Preparation

*C. albicans* strain SC5314 was obtained from the China Medical Fungal Culture Collection Center. For yeast‐phase expansion, *C. albicans* was cultured in yeast extract‐peptone‐dextrose (YPD) medium (OXOID, UK) in a rotary shaker at 30°C and 180 rpm for 12 h, washed in PBS, and resuspended to the required concentration.

### 4.4 Animal

Wild‐type (WT) C57BL/6J mice were purchased from Jiangsu Jicui Yaokang Biotechnology (Nanjing, China). MC‐deficient Kit^W‐sh/W‐sh^ mice were obtained from the Model Animal Research Center of Nanjing University (Nanjing, China). IL‐6 knockout (IL‐6^−/−^) mice were purchased from Jiangsu Provincial Southern Model Biotechnology (Nanjing, China).

For all in vivo experiments, male and female mice were used at an approximate ratio of 3:2, aged 6–8 weeks and weighing 18–22 g at the onset of experiments. Mice were housed in a specific pathogen‐free (SPF) environment with a 12‐h light/dark cycle, an indoor temperature maintained at 22–25°C, and a relative humidity of 30%‐70%. The animals had ad libitum access to standard laboratory chow and sterile water.

All animal husbandry and experimental procedures complied with the NIH Guide for the Care and Use of Laboratory Animals. Animal health and behavior were monitored daily by trained personnel. To minimize pain and distress during procedures such as dorsal shaving and *C. albicans* inoculation, mice were anesthetized with inhaled isoflurane (2%–3% for induction, 1.5%–2% for maintenance). At the end of experiments, or upon reaching predefined humane endpoints, mice were euthanized by CO_2_ asphyxiation followed by cervical dislocation.

### 4.5 Cutaneous *Candida* Infection

The cutaneous *C. albicans* infection model was established as previously described, with minor modifications [34, 35]. Briefly, the backs of mice were first shaved. Under stable anesthesia, the dorsal skin of the mice was stripped with Tegaderm (3 m) tape to disrupt the skin barrier. To ensure a standardized and quantitative degree of skin damage across all animals, the tape stripping procedure was uniformly repeated 6 times until a specific macroscopic endpoint was reached. The standard for optimal barrier disruption was defined as the targeted skin area exhibiting a glistening and mildly erythematous appearance (indicative of stratum corneum removal), while strictly ensuring the absence of any macroscopic bleeding or deep dermal injury. Then, 50 µL of 2 × 10^8^ CFU/mL *C. albicans* [36] was evenly applied to the backs of the mice, and the changes in the dorsal skin of the mice were observed.

For IL‐6 intervention experiments, mice received an intradermal injection of recombinant mouse IL‐6 (rmIL‐6, 200 ng; BioLegend, USA) or an IL‐6‐neutralizing antibody (anti‐IL‐6, 20 µg; BioLegend, USA) in 50 µL of PBS at the intended infection site, 1 h prior to *C. albicans* application. Control animals received an equal volume of sterile PBS.

### 4.6 Mast‐Cell Engraftment of Kit^W‐sh/W‐sh^ Back Skin

For the isolation and induction of bone marrow‐derived mast cells (BMMCs), detailed procedures can refer to our previous research methods [37]. In brief, bone marrow was flushed from the tibias and femurs of 4‐week‐old C57BL/6J mice, and cells were cultured in RPMI‐1640 medium (Gibco, USA) supplemented with 10% heat‐inactivated fetal bovine serum (Gibco, USA), 1% penicillin‐streptomycin (Gibco, USA), 10 ng/mL stem cell factor (SCF; BioLegend, USA), and 10 ng/mL interleukin‐3 (IL‐3; BioLegend, USA). Medium was refreshed every 7 days for 4 weeks. BMMC purity was assessed by flow cytometry, and cultures with ≥95% c‐Kit^+^FcεRIα^+^ cells were considered mature and used for downstream experiments.

For adoptive transfer experiments, mature BMMCs were harvested from 2 to 3 donor mice of the same genotype (WT or IL‐6^−/−^) and pooled prior to injection to minimize inter‐donor variability. Pooled BMMCs (4×10^6^ cells per recipient, corresponding to 10^6^ cells/cm^2^ over a 4 cm^2^ dorsal area) were intradermally injected into Kit^W‐sh/W‐sh^ recipients at 8 evenly distributed sites (5×10^5^ BMMCs in 25 µL PBS per site). Each recipient mouse was treated as one independent biological replicate, and all downstream assays (qRT‐PCR, ELISA) were performed in technical triplicate on individual samples to ensure analytical precision. Four weeks after adoptive transfer, successful reconstitution of cutaneous mast cells was confirmed by toluidine blue staining of the grafted skin.

### 4.7 In Vitro *C. albicans* Infection Assays

Primary mouse epidermal keratinocytes (MEKs) were isolated as previously described [38]. Briefly, epidermal sheets separated from newborn mouse skin using dispase II (Sigma‐Aldrich, USA) were further digested with trypsin into single cells, which were then cultured in Keratinocyte Medium (ScienCell, USA) supplemented with 1% penicillin‐streptomycin‐nystatin solution.

For co‐culture experiments, MEKs and BMMCs were seeded into 6‐well plates at MEK:BMMC ratios of 1:1, 2:1, and 1:2 (total cell number kept constant across conditions). Once MEKs had adhered, cells were infected with *C. albicans* at a multiplicity of infection (MOI) of 1 and co‐incubated for 4 h at 37°C. After incubation, cells and supernatants were harvested, thoroughly mixed, serially diluted in sterile PBS, and plated onto Sabouraud Dextrose Agar (SDA). Colonies were enumerated after 18 to 32 h of incubation at 37°C.

For cytokine intervention, recombinant mouse IL‐6 (rmIL‐6, 50 ng/mL or as indicated) or an IL‐6‐neutralizing antibody (anti‐IL‐6, 25 ng/mL) was added to the co‐culture medium 1 h prior to *C. albicans* infection.

For physical separation experiments, BMMCs and MEKs were cultured in the upper and lower chambers of Transwell inserts with a 0.4‐µm pore size (Corning, USA), respectively, and infection was performed as described above.

### 4.8 Acquisition of Skin Single‐cell Suspension and Flow Cytometry Analysis

Skin tissues were processed using our previously described method with modifications [39]. Epidermal and dermal layers were separated using dispase II; dermal tissues were further digested with collagenase IV (Sigma, USA) and DNase (Sigma, USA) to obtain single‐cell suspensions.

Single‐cell suspensions were then incubated with the appropriate fluorochrome‐conjugated antibody cocktails. Mast cells were stained with PE/Cy7‐CD45 (BioLegend, USA; Cat.103114), PerCP/Cy5.5‐CD11b (BioLegend, USA; Cat.101228), FITC‐c‐Kit (BioLegend, USA; Cat.105811) and PE‐FcεRIα (BioLegend, USA; Cat.134307); neutrophils were stained with PE/Cy7‐CD45, PerCP/Cy5.5‐CD11b and PE‐Ly6G (BD Biosciences, USA; Cat.551461). To ensure rigorous compensation and unbiased gating, single‐stained compensation controls were prepared for each fluorochrome using single‐stained cells. Then, Fluorescence Minus One (FMO) controls were used as the gold standard for objective gating: FMO–c‐Kit and FMO–FcεRIα controls defined the positive/negative boundaries of the c‐Kit^+^ FcεRIα^+^ MCs population, and an FMO–Ly6G control defined the boundary of the Ly6G^+^ neutrophil population. Cells were then acquired on a FACS Aria II flow cytometer (BD Biosciences, USA), and the data were analyzed using FlowJo software (BD Biosciences, USA).

### 4.9 Multiplex Secretome Analysis

Cell supernatants were collected according to the requirements of the kit from ABclonal Technology Co., Ltd. (Hangzhou, China). Fluorescently encoded microspheres (ABplex Mouse Multiplex Custom Detection Plate, ABclonal Technology Co., Ltd.) were mixed, and the supernatants were added for detection and analysis according to the manufacturer's instructions.

### 4.10 Small Interfering RNA Transfection

Cultured MEKs were transfected with mouse NF‐κB2 siRNA and STAT3 siRNA or a scrambled negative control siRNA (GenePharma, Shanghai, China) at a final concentration of 12.5 nM, using Lipofectamine RNAiMAX (Invitrogen, USA) according to the manufacturer's instructions. Three independent siRNA sequences were tested for each target gene to control for off‐target effects; sequences are provided in Table S3. Knockdown efficiency was validated by qRT‐PCR and western blot before downstream experiments.

### 4.11 Histology, Toluidine Blue, PAS Staining and Semi‐Quantitative Histopathological Scoring System

Infected site biopsies were fixed in 4% paraformaldehyde (PBS, pH 7.4) at 4°C for 24 h, processed into 4‐µm paraffin sections, and mounted on poly‐L‐lysine slides.​Deparaffinized sections were stained with 0.1% toluidine blue (pH 2.5) for 10 min, differentiated, and mounted. Mast cells showed purple‐blue granules (light microscopy).​

Sections were treated with 0.5% periodic acid (10 min), incubated with Schiff's reagent (dark, 15 min), counterstained with hematoxylin, and mounted. Fungal structures appeared magenta.​ Stained sections were imaged digitally; mast cell count and fungal burden were quantified via CaseViewer by blinded observers.​

To quantify the severity of cutaneous *C. albicans* infection, a composite histopathological scoring system (0–6) was employed, adapted from established models [40, 41]. Evaluation focused on two core parameters, each graded from 0 to 3: Inflammatory Cell Infiltration Score: 0: Absence of inflammatory cells. 1: Scattered cells restricted to superficial dermal perivascular areas. 2: Distinct aggregation with intraepidermal abscesses (e.g., Munro microabscesses) or significant spongiosis. 3: Diffuse, high‐density infiltration forming a continuous inflammatory band. Tissue Injury Score:0: Intact dermo‐epidermal junction. 1: Minor edema or vasodilation within a fundamentally intact architecture. 2: Reactive changes (epidermal hyperplasia, acanthosis, hyperkeratosis, or microvacuolization). 3: Substantial destruction (necrosis, ulceration, or pseudoepitheliomatous hyperplasia) extending into the deep dermis. Samples were stratified into mild infection (composite score ≤ 3) and severe infection (composite score > 3) for downstream analysis. All sections were independently scored by two pathologists blinded to group identity; in case of discordance, consensus was reached through joint review.

### 4.12 RNA Extraction and Quantitative Reverse Transcription PCR (qRT‐PCR)

For detailed information, please refer to our previous research [39]. Total RNA was extracted from cells or tissue homogenates with TRIzol reagent (Invitrogen, Carlsbad, CA, USA). cDNA was synthesized using HiScript III RT SuperMix for qPCR with gDNA wiper (Vazyme, Nanjing, China) according to the manufacturer's protocol. Quantitative PCR was performed on a QuantStudio 5 using SYBR Green Pro Taq HS Premix (Selleck, Shanghai, China). Relative mRNA expression was calculated by the 2^−ΔΔCt^ method with GAPDH as the endogenous reference gene, which was chosen based on its well‐documented stability in murine skin and cultured keratinocytes under inflammatory conditions and was further confirmed by consistent raw Ct values across all experimental conditions in this study. Primer sequences are listed in Table S3.

### 4.13 Protein Extraction and Western Blotting (WB)

Cells and tissue samples were lysed in RIPA buffer supplemented with phosphatase and protease inhibitors (Beyotime, Shanghai, China) at 4°C for 30 min, followed by brief sonication. Lysates were clarified by centrifugation at 12,000 × g for 15 min at 4°C. Protein concentrations were determined using a BCA assay kit (Beyotime, Shanghai, China). Equal amounts of protein (20‐40 µg per lane) were mixed with SDS loading buffer (Beyotime, Shanghai, China), denatured at 95°C for 5 min, resolved on 12.5% SDS‐PAGE gels at 80–120 V, and transferred onto polyvinylidene fluoride (PVDF) membranes (Bio‐Rad, USA). Membranes were blocked with 5% non‐fat milk in TBST for 1 h at room temperature, incubated with primary antibodies overnight at 4°C, washed in TBST, and incubated with HRP‐conjugated secondary antibodies for 1 h at room temperature. Protein bands were visualized with an ECL detection kit (Bio‐Rad, USA) and imaged using Amersham ImageQuant 800 system (Cytiva, Japan).

### 4.14 ELISA

ELISA kits for mouse CRAMP, β‐defensin‐2 (BD‐2), β‐defensin‐3 (BD‐3), IL‐6, and CXCL2 were purchased from MultiSciences Biotechnology (Hangzhou, China). Culture supernatants and skin tissue homogenates were analyzed according to the manufacturer's instructions. Optical density was measured at 450 nm on a microplate reader, and target protein concentrations were calculated from four‐parameter logistic (4‐PL) standard curves. The detection limit of each assay, defined as the mean OD of the zero standard plus two standard deviations, corresponded to the following sensitivities: CRAMP (0.1 ng/mL), BD‐2 (1 pg/mL), BD‐3 (1 pg/mL), IL‐6 (1 pg/mL), and CXCL2 (1 pg/mL). All reported concentrations fell within the validated dynamic range of the corresponding standard curve; samples exceeding the upper limit of quantification were diluted in the kit‐supplied sample diluent and re‐assayed.

### 4.15 TSA‐based Immunofluorescence Staining and Quantitative Analysis

To assess in situ cytokine expression, immunofluorescence (IF) staining was performed using Tyramide Signal Amplification (TSA) on 4‐µm FFPE skin sections. Briefly, after deparaffinization, rehydration, and microwave‐based antigen retrieval (pH 6.0), sections were treated with 3% H_2_O_2_ and blocked with 5% BSA. Parallel sections were incubated overnight at 4°C with primary antibodies against CXCL2 (Abcam, ab317569, 1:200) or IL‐6 (Bioss, bs‐0782R, 1:200). Signals were developed using HRP‐conjugated secondary antibodies and TSA‐Cy3, followed by DAPI counterstaining and anti‐fade mounting.

Confocal images (Olympus FV3000) were acquired under identical settings (laser power, exposure, and gain) to ensure comparability. Using Fiji/ImageJ, the corrected Mean Fluorescence Intensity (MFI) was calculated by subtracting the mean gray value of a cell‐free background area from that of three dermo‐epidermal junction ROIs per section. MFI values were averaged per patient to represent a single biological replicate.

### 4.16 Statistical Analyses

Data are presented as mean±standard deviation (SD) from at least three independent biological replicates, with “n” denoting the number of mice per group for in vivo studies. Technical replicates were averaged per biological sample prior to analysis to avoid pseudo‐replication. Normality and homoscedasticity were assessed by the Shapiro‐Wilk and Brown‐Forsythe tests, respectively. For two‐group comparisons, Student's unpaired two‐tailed t‐test was used; Welch's correction was applied when variances were unequal. For three or more groups, one‐way ANOVA with Tukey's post‐hoc test was used, or the Brown‐Forsythe and Welch ANOVA when assumptions were violated. All statistical analyses were performed in GraphPad Prism 9.0 (GraphPad Software, USA). Significance: *p < 0.05, **p < 0.01, ***p < 0.001, ****p < 0.0001.

## Author Contributions

Conceptualization, ML, XC, YY; Methodology, MM, JW, ZD; Investigation, MM, JW, YC, SC, CL; Resources, JZ, XZ, SG; Data curation, XZ, CL; Writing – original draft, YY; Writing – review and editing, ML, XC, NL; Supervision, ML, XC, NL.

## Conflicts of Interest

The authors declare no competing interests.

## Supporting information




**Supporting File 1**: advs75505‐sup‐0001‐SuppMat.pdf.

**Supporting File 2**: advs75505‐sup‐0002‐DataFile.pdf.

## Data Availability

The data that support the findings of this study are available from the corresponding author upon reasonable request.
