# Supplementary material for: A Keratinocyte‐Mast Cell NF‐κB2/CXCL2/IL‐6 Amplification Loop Enhances Cutaneous Antifungal Defense Against C. albicans
Source: Adv Sci (Weinh). 2026 May 4;13(41):e20409. doi: 10.1002/advs.202520409 (PMC13335439; doi:10.1002/advs.202520409)
Supplement: Supplementary file 1 — Supporting File 1: advs75505‐sup‐0001‐SuppMat.pdf. [file ADVS-13-e20409-s001.pdf]

Supplementary Materials for

Amplification Loop between Keratinocytes and Mast cells Enhances Antifungal Defense against *C. albicans* Skin Infection

Authors: Yan Yuan[1], Manyun Mao[1], Jiaan Zhang[1], Zhimin Duan[1], JiaNing Wang[1], Yujie Chen[1], Sihan Chen[1], Chuting Liang[1], Xvyue Zhou[1], Songmei Geng[2], Min Li[1,3*], Xu Chen[1,3*], Ni Lian[1*]

[1]Jiangsu Provincial Key Laboratory of Dermatology, Hospital for Skin Diseases, Institute of Dermatology, Chinese Academy of Medical Sciences & Peking Union Medical College, Nanjing, 210042, China.

[2]Department of Dermatology, The Second Affiliated Hospital of Xi'an Jiaotong University, No. 157 Xiwu Road, Xi'an, 710004, China

[3]Center for Global Health, School of Public Health, Nanjing Medical University, Nanjing, China.

[*]Correspondence: Min Li, E-mail: limin@pumcderm.cams.cn, Xu Chen, E-mail: chenx@pumcderm.cams.cn, Ni Lian, E-mail: lianni@aliyun.com.

The PDF file includes: Figure S1 to S6; Table S1 to S3

Figure S1

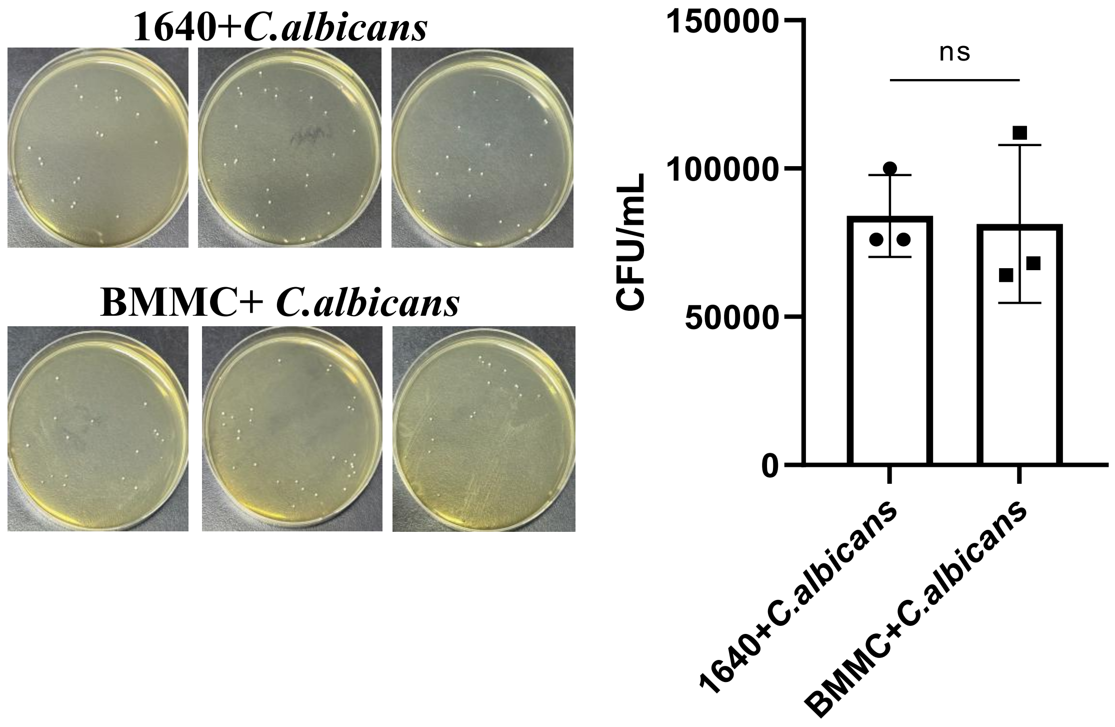

Figure S1. BMMCs alone do not restrict *C. albicans* growth in vitro. Representative CFU images (left) and quantification (right) of C. albicans in RPMI-1640 medium alone or co-cultured with BMMCs (MOI = 1, 4 hours) (n=3). Data are presented as mean ± SD. ns, not significant

Figure S2

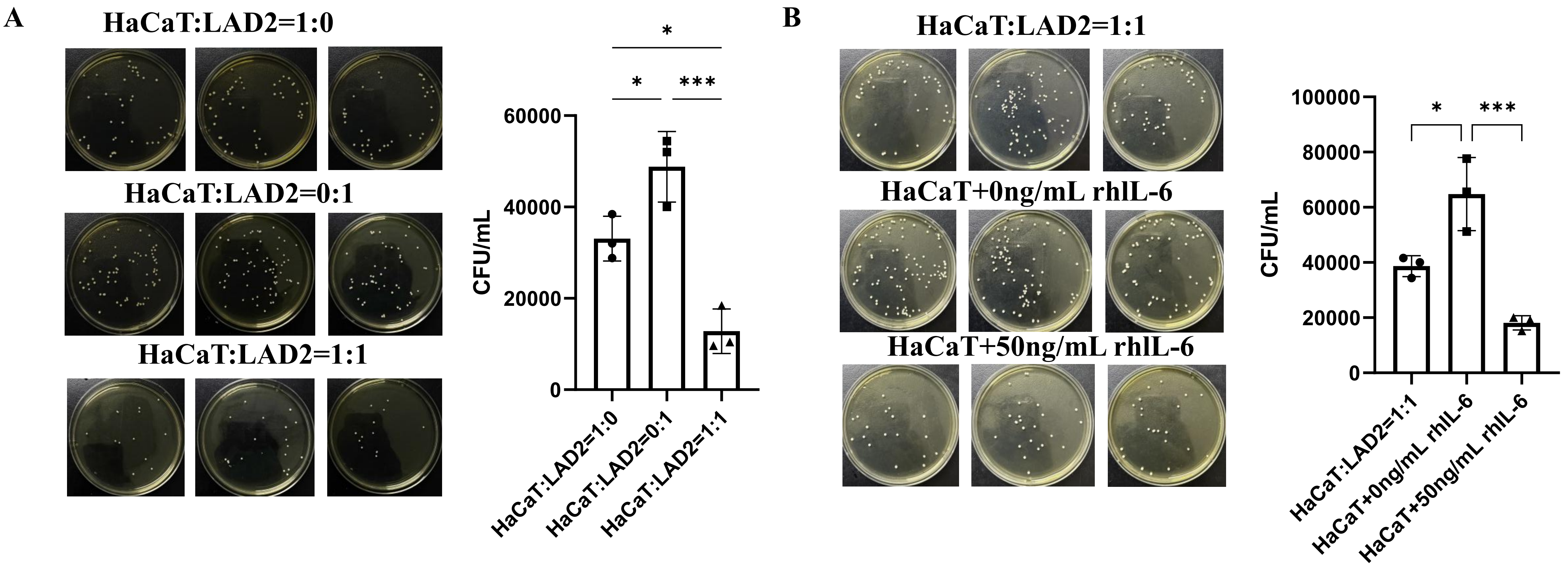

Figure S2. MC-KC synergy and IL-6-mediated antifungal activity in human cells.

A. Representative CFU images (left) and quantification (right) of C. albicans in HaCaT monoculture (HaCaT: LAD2 = 1:0), LAD2 monoculture (HaCaT:LAD2 = 0:1), or HaCaT-LAD2 co-culture (HaCaT: LAD2 = 1:1). n = 3 per group. B. Representative CFU images (left) and quantification (right) of C. albicans in HaCaT+LAD2 (1:1) co-culture, HaCaT+0 ng/mL rhIL-6, and HaCaT+50 ng/mL rhIL-6 conditions (n=3). Data are presented as mean ± SD. *$P < 0.05$, ***$P < 0.001$ (one-way ANOVA followed by Tukey's multiple comparisons test).

FigureS3

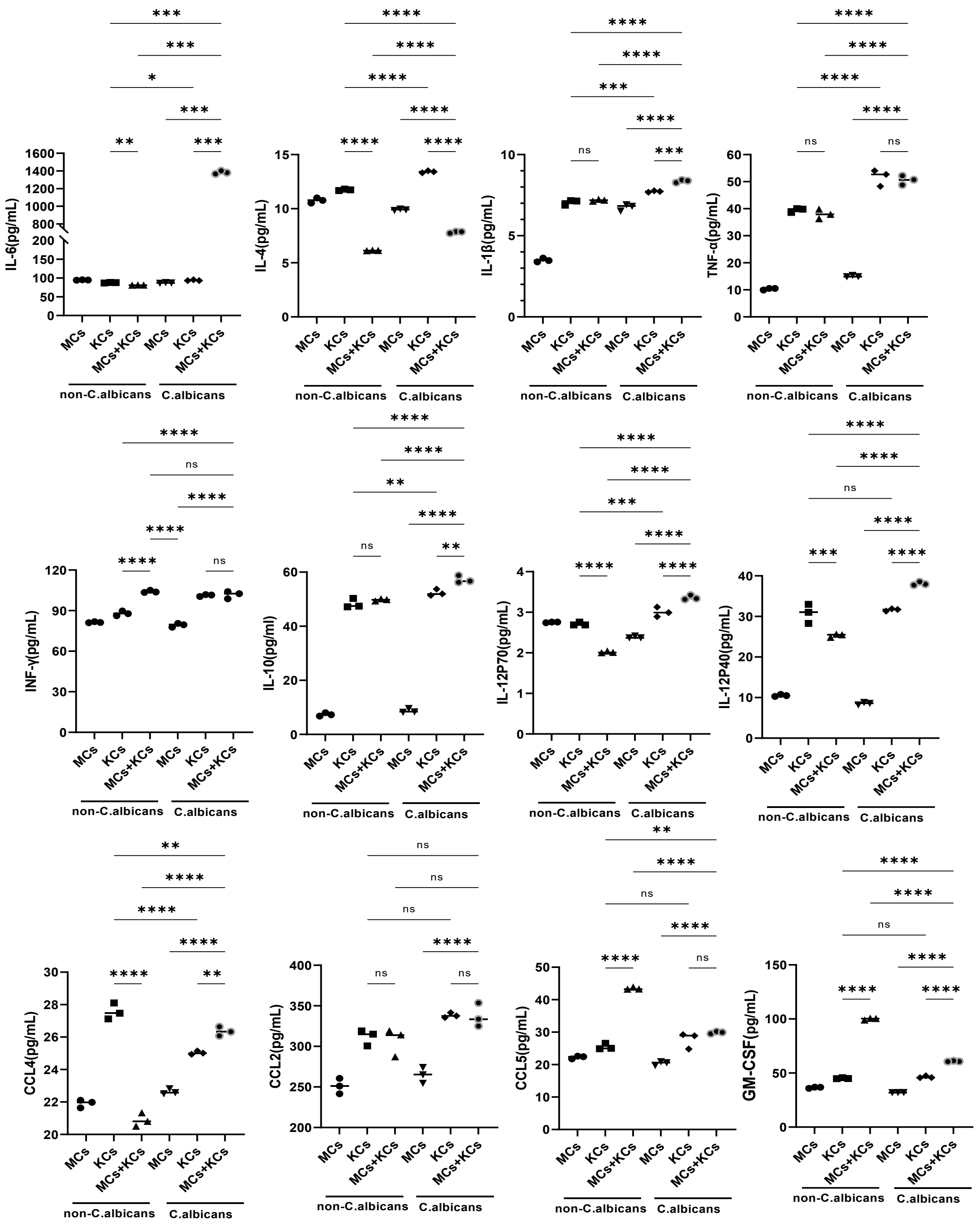

Figure S3. Multiplex cytokine profiling of MEK-BMMC co-cultures during *C. albicans* infection. Supernatants were collected from MCs alone, KCs alone, and MC-KC co-cultures (1:1 ratio), with or without C. albicans infection (MOI = 1, 4 hours). Concentrations of IL-6, IL-4, IL-1β, TNF-α, IFN-γ, IL-10, IL-12p70, IL-12p40, CCL4, CCL2, CCL5, and GM-CSF were measured by multiplex secretome analysis (n=3). Data are presented as mean ± SD. *P < 0.05, **P < 0.01, ***P < 0.001, ****P < 0.0001; ns, not significant.

Figure S4

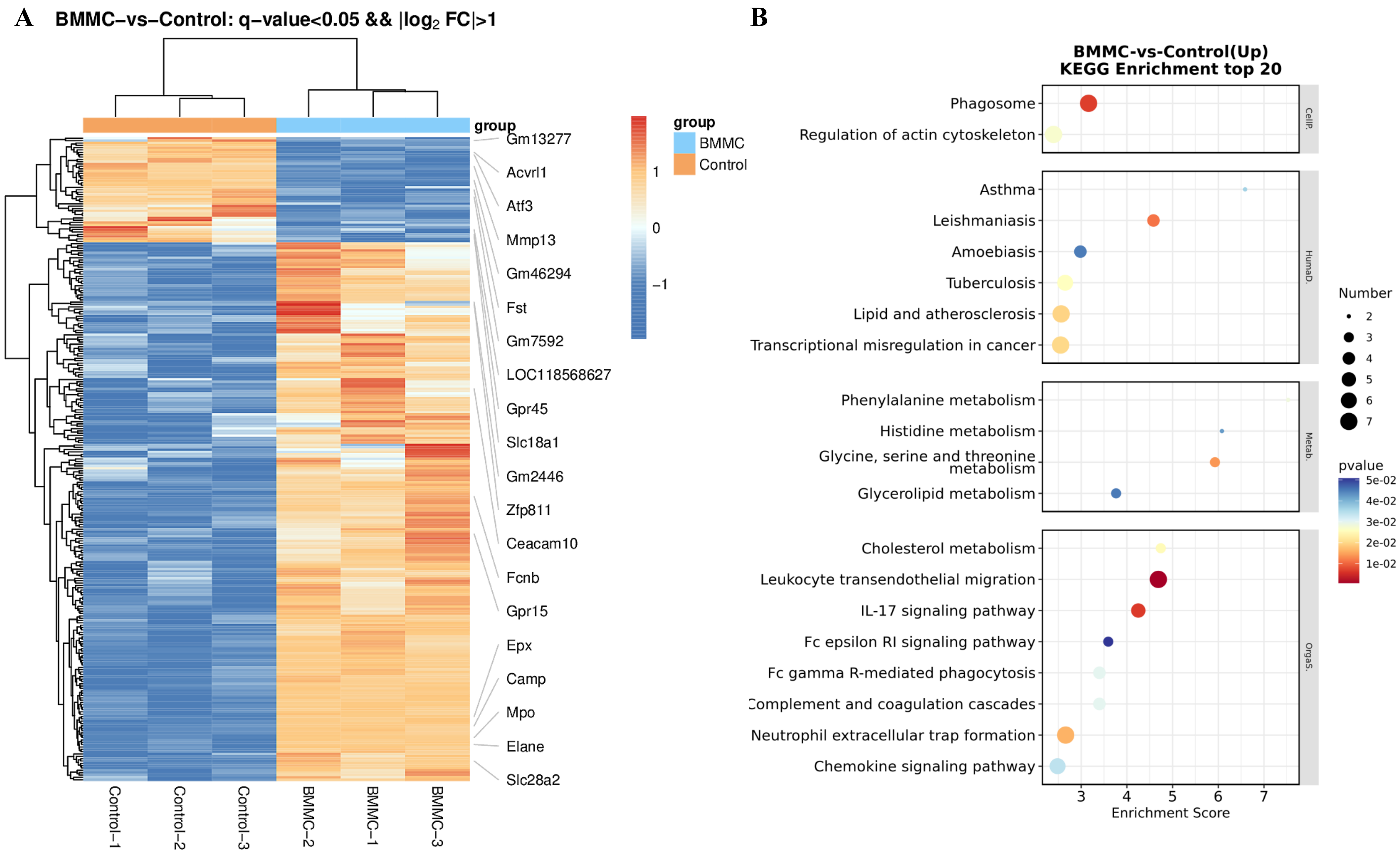

Figure S4. Transcriptomic profiling of BMMCs after CXCL2 stimulation.

A. Representative CFU images (left) and quantification (right) of C. albicans in HaCaT monoculture (HaCaT:LAD2 = 1:0), LAD2 monoculture (HaCaT:LAD2 = 0:1), or HaCaT-LAD2 co-culture (HaCaT:LAD2 = 1:1). n = 3 per group. B. Representative CFU images (left) and quantification (right) of C. albicans in HaCaT+LAD2 (1:1) co-culture, HaCaT+0 ng/mL rhIL-6, and HaCaT+50 ng/mL rhIL-6 conditions (n=3). Data are presented as mean ± SD. *P < 0.05, ***P < 0.001 (one-way ANOVA followed by Tukey's multiple comparisons test).

Figure S5

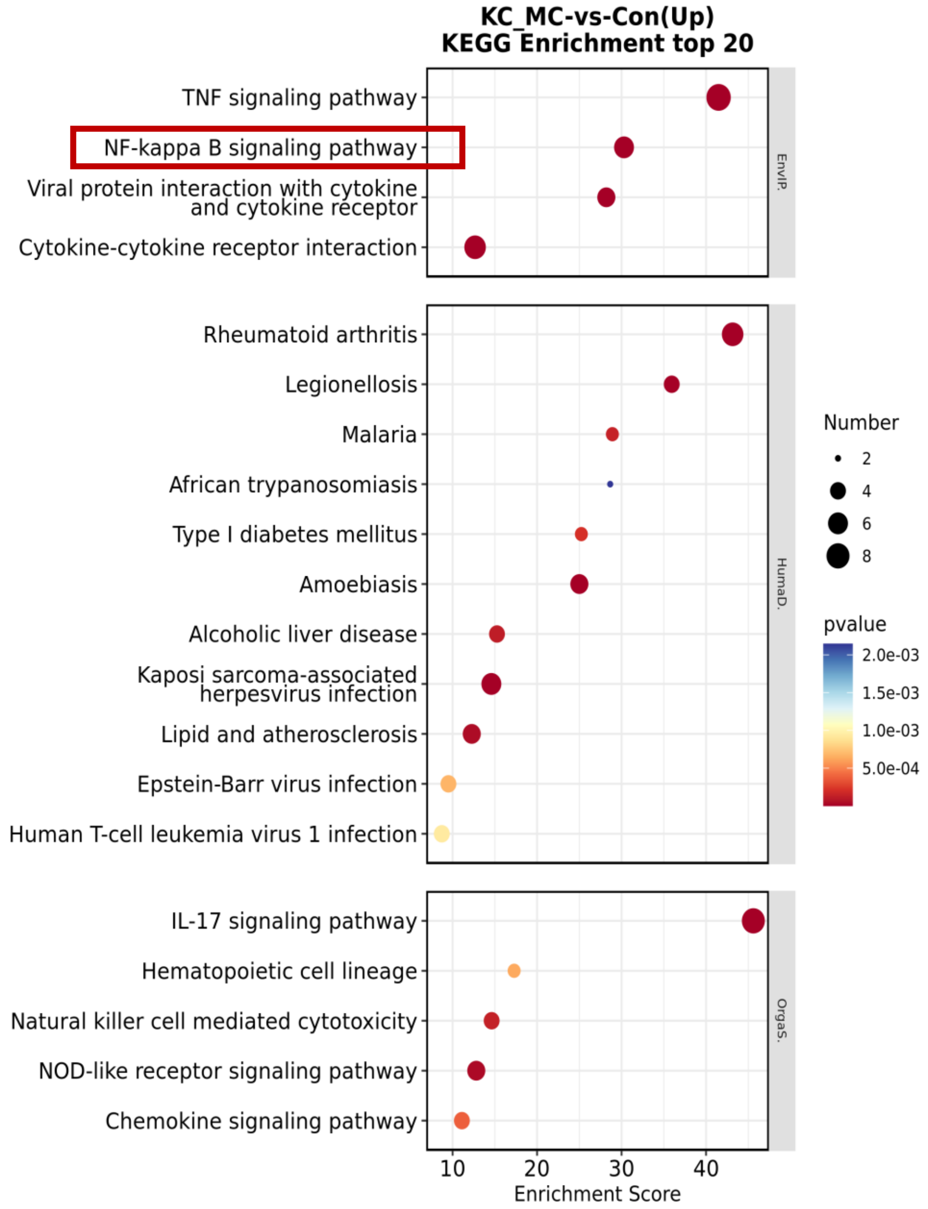

Figure S5. KEGG pathway enrichment analysis of MEKs in MEK-BMMC co-cultures. Top 20 KEGG-enriched pathways among genes upregulated in MEKs from MEK+BMMC+C. albicans co-cultures compared with MEK+C. albicans monocultures. The NF-κB signaling pathway is highlighted in red (n=3).

Figure S6

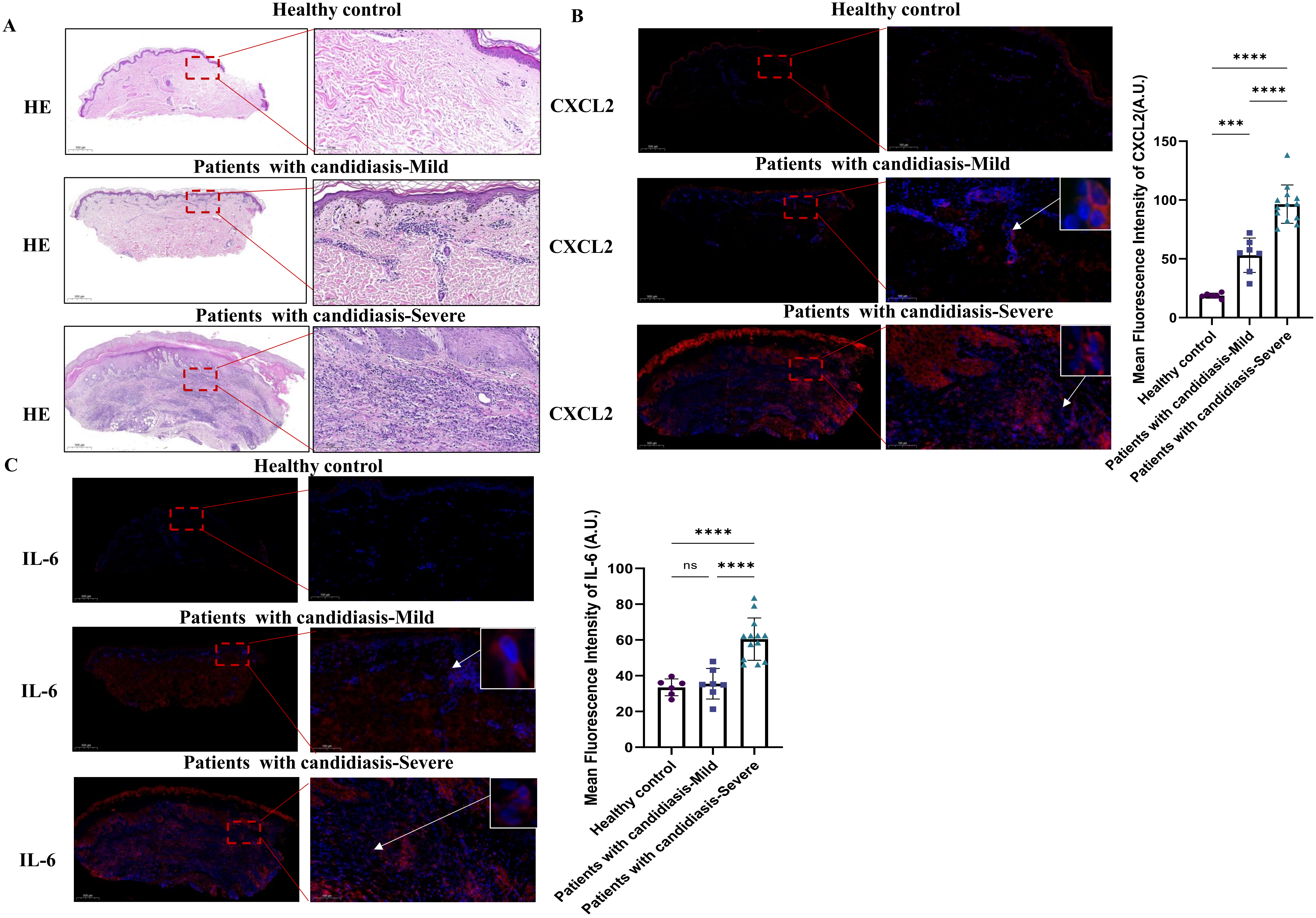

Figure S6. CXCL2 and IL-6 expression correlate with infection severity in cutaneous candidiasis.

A. Representative H&E-stained skin sections from healthy controls (top), patients with mild cutaneous candidiasis (middle), and patients with severe cutaneous candidiasis (bottom). Infection severity was stratified by the composite histopathological score (see Methods). Scale bar: 500 μm (overview), 100 μm (inset). B. Representative TSA-immunofluorescence staining of CXCL2 (red) with DAPI nuclear counterstain (blue) in the three groups (left); quantification of CXCL2 mean fluorescence intensity (MFI, right). Scale bar: 500 μm (overview), 100 μm (inset). C. Representative TSA-immunofluorescence staining of IL-6 (red) with DAPI nuclear counterstain (blue) in the three groups (left); quantification of IL-6 MFI (right). Scale bar: 500 μm (overview), 100 μm (inset). Healthy controls, n = 6; mild candidiasis, n=7; severe candidiasis, n=13. Data are presented as mean ± SD. ***$P < 0.001$, ****$P < 0.0001$; ns, not significant.

Table S1 Demographic information of the initial cohort.

Samples	Tissue sampling site	Sex	Age
Patient-1	Trunk	Female	50
Patient-2	Trunk	Female	63
Patient-3	Extremities	Female	65
Patient-4	Extremities	Female	69
Patient-5	Extremities	Male	64
Patient-6	Extremities	Male	60
Patient-7	Face	Male	53
Patient-8	Extremities	Female	57
Patient-9	Extremities	Male	20
Patient-10	Extremities	Female	76
Healthy control-1	Face	Male	30
Healthy control-2	Extremities	Female	56
Healthy control-3	Trunk	Female	40
Healthy control-4	Extremities	Female	71
Healthy control-5	Extremities	Female	61
Healthy control-6	Trunk	Male	22

Table S2 Demographic information of the expanded cohort.

Samples	Tissue sampling site	Sex	Age
Patient-1	Extremities	Male	64
Patient-2	Extremities	Male	60
Patient-3	Face	Male	53
Patient-4	Extremities	Female	57
Patient-5	Extremities	Male	20
Patient-6	Extremities	Female	76
Patient-7	Trunk	Male	88
Patient-8	Extremities	Male	59
Patient-9	Extremities	Female	78
Patient-10	Face	Male	40
Patient-11	Extremities	Male	73
Patient-12	Extremities	Female	83
Patient-13	Extremities	Female	60
Patient-14	Extremities	Female	47
Patient-15	Trunk	Male	65
Patient-16	Extremities	Male	60
Patient-17	Extremities	Male	78
Patient-18	Face	Female	70
Patient-19	Extremities	Female	64
Patient-20	Extremities	Male	50

Table S3 The primer sequences for the genes.

Species	Gene name	sense (5'-3')	antisense (5'-3')
Mouse	CXCL2	TCCAGACTCCAGCCACACTTC	AGCAGCAGCAGGACCAGTG
Mouse	TNF	CACGCTCTTCTGTCTACTGAACTTC	CTTGGTGGTTTGTGAGTGTGAGG
Mouse	CSF-2	CCCACCCGCTCACCCATC	AGACGACTTCTACCTCTTCATTCAAC
Mouse	IL-6	CCGCTATGAAGTTCCTCTC	GGTATCCTCTGTGAAGTCTC
Mouse	CCL2	CAGCCAGATGCAGTTAACGC	GCTGCTGGTGATCCTCTTGT
Mouse	CCL4	CTAACCCCGAGCAACACCAT	ATTGGTGCTGAGAACCCTGG
Mouse	CCL5	AGACAGCACATGCATCTCCC	GTCCGAGCCATATGGTGAGG
Mouse	IL-12B	TTGCCATCGTTTTGCTGGTG	CACTGTTTCTCCAGGGG CAT
Mouse	IFN-γ	GCAAGGCGAAAAAGGATGCA	CGACTCCTTTTCCGCTTCCT
Mouse	CAMP	ACACCAATCTCTACCGTCTCCTG	CGCTCTGCCTTGCCACATAC
Mouse	DRFB2	GGGATGCTGCAATTTTGTTGGAG	TCTGGCAGAAGGAGGACAAGTG
Mouse	DRFB3	TTCTCCTGGTGCTGCTGTCTC	ATCTGACGAGTGTTGCCAATGC
Mouse	IL-1β	CTTCAGGCAGGCAGTATC	CAGCAGGTTATCATCATCATC
Mouse	GAPDH	GGTTGTCTCCTGCGACTTCA	TGGTCCAGGGTTTCTTACTCC
Mouse	NF-κB2-1137	CUCCCACAGACGUUCAUAATT	UUAUGAACGUCUGUGGGAGTT
Mouse	NF-κB2-1809	CUGGUCAUUGAGCAGAUTT	AUCUGCUCAAUGACCAGTT
Mouse	NF-κB2-452	CGAGGCUUCAGAUUUCGAUTT	AUCGAAAUCUGAAGCCUCGTT
Mouse	Stat3-403	GCAAGAGUCCAAUGUCCUCTT	GAGGACAUUGGACUCUUGCTT
Mouse	Stat3-1110	GGAUCGUGGAGCUGUUCAGTT	CUGAACAGCUCCACGAUCCTT
Mouse	Stat3-1450	AGGCCGUGCCAAUUGUGAUTT	AUCACAAUUGGCACGGCCUTT
