# Supplementary figures and images for: A Keratinocyte‐Mast Cell NF‐κB2/CXCL2/IL‐6 Amplification Loop Enhances Cutaneous Antifungal Defense Against C. albicans
Source: Adv Sci (Weinh). 2026 May 4;13(41):e20409. doi: 10.1002/advs.202520409 (PMC13335439; doi:10.1002/advs.202520409)

Figure 7A

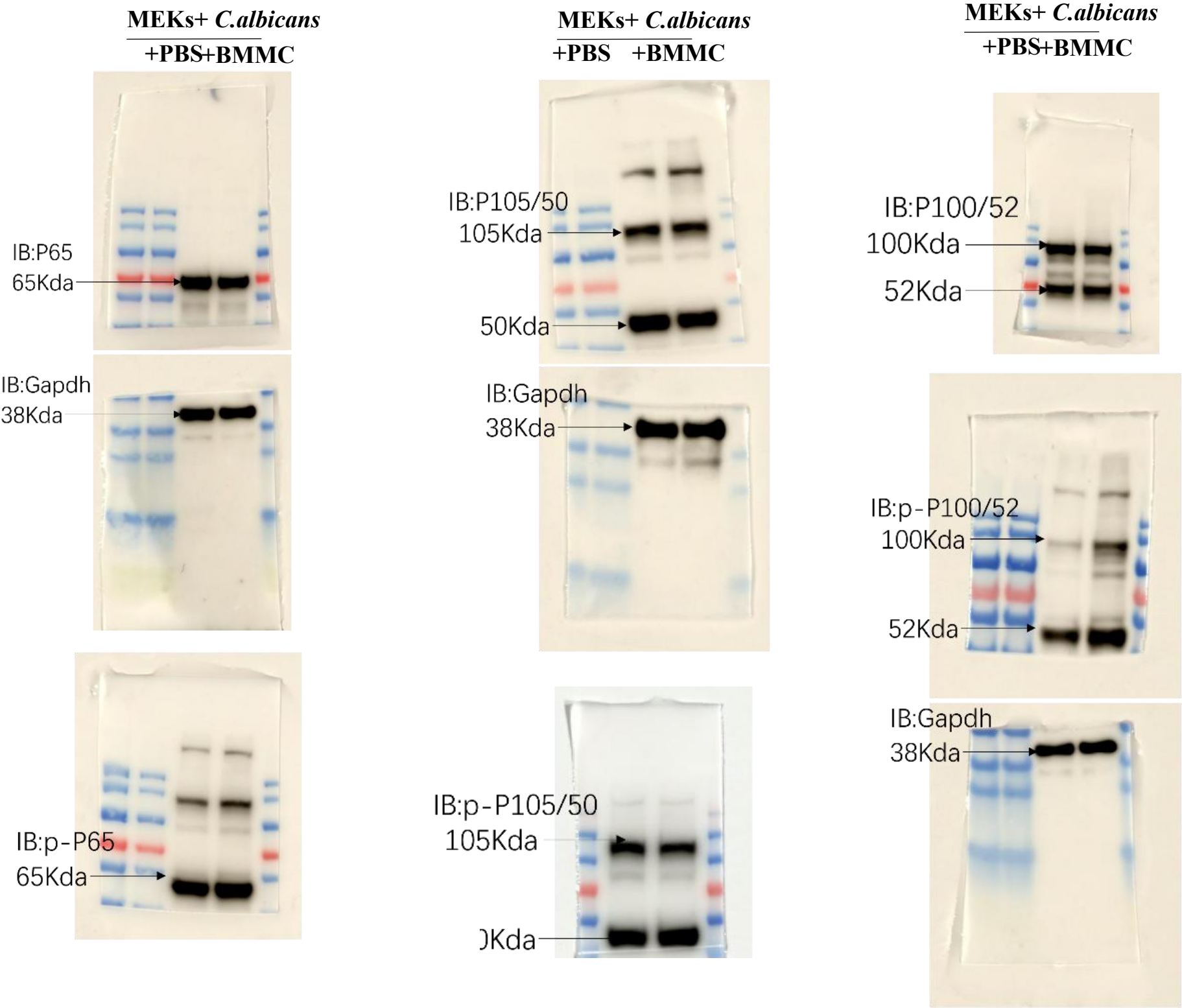

Figure 7C

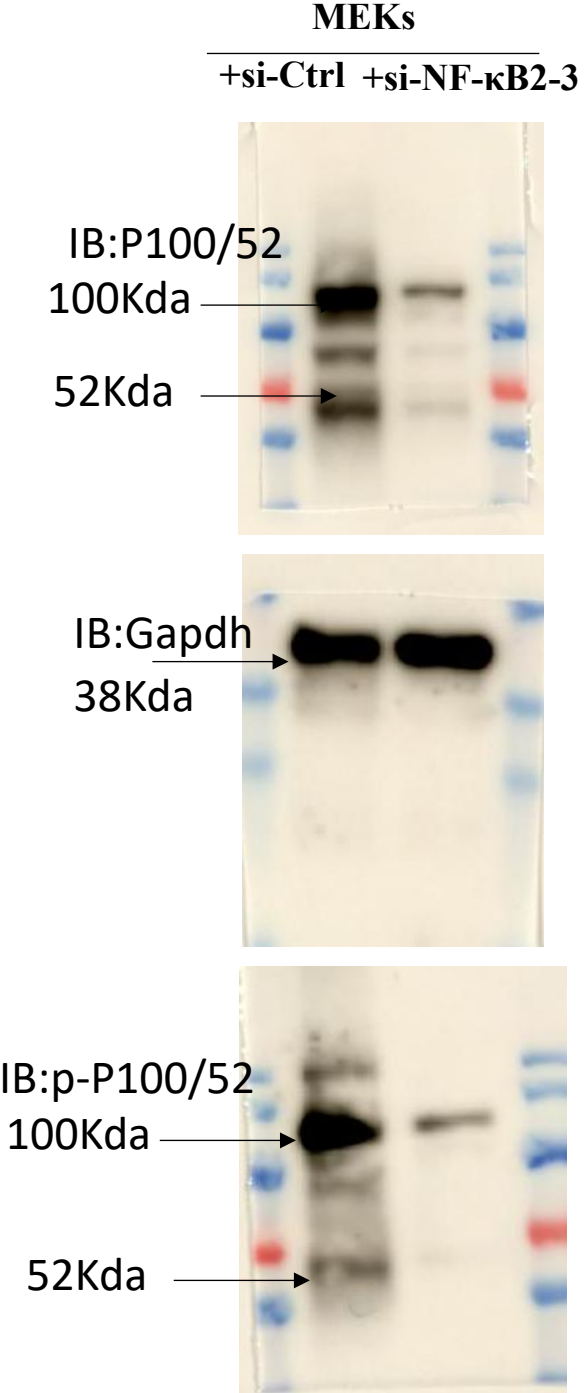

Figure 7K

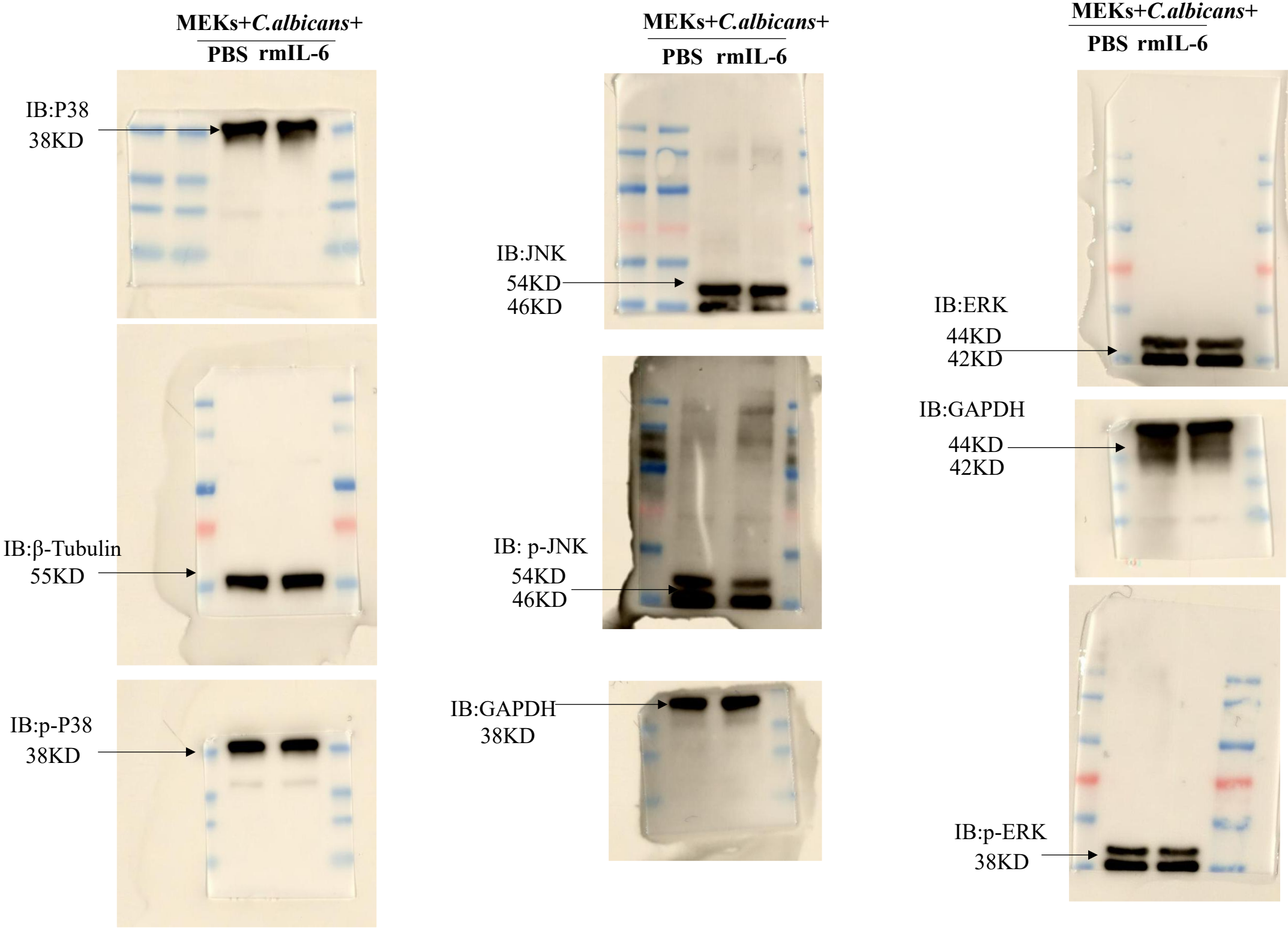

Figure 7L

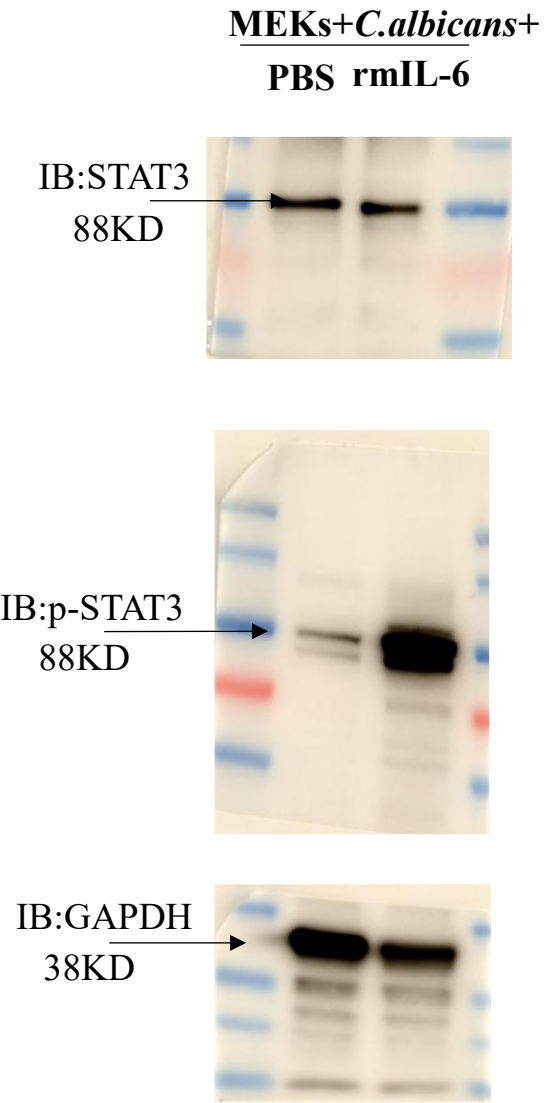

Figure 7N

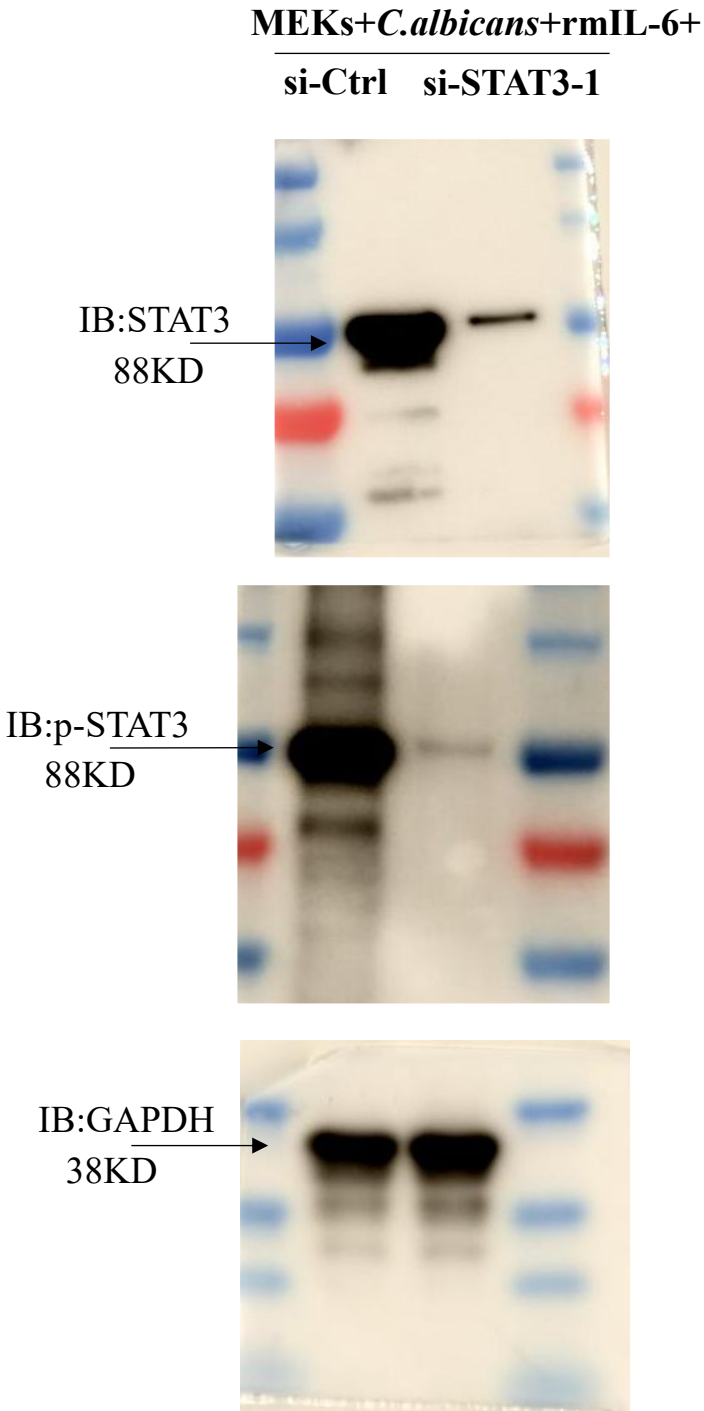

Supplement: Supplementary file 2 — Supporting File 2: advs75505‐sup‐0002‐DataFile.pdf. [file ADVS-13-e20409-s002.pdf]
